# Hi-C Metagenome Deconvolution of Double-Crested Cormorant (*Nannopterum auritum*) Fecal Samples Demonstrates Feasibility of Linking Microbial Genomes, AMR Genes, and Mobile Elements in Avian Microbiomes

**DOI:** 10.3390/microorganisms14061198

**Published:** 2026-05-26

**Authors:** Sydney N. O’Donald, Fenny Patel, Patricia Keen, Larry A. Hanson, Frederick Cunningham, Mark L. Lawrence, Hasan C. Tekedar

**Affiliations:** 1 Department of Comparative Biomedical Sciences, College of Veterinary Medicine, Mississippi State University, Starkville, MS 39762, USA; 2 Department of Energy Management, New York Institute of Technology, Vancouver, BC V6B 4P8, Canada; 3 United States Department of Agriculture—Animal and Plant Health Inspection Service, Wildlife Services, National Wildlife Research Center—Mississippi Field Station, Starkville, MS 39762, USA; 4 Global Center for Aquatic Health and Food Security, Mississippi State University, Starkville, MS 39762, USA

**Keywords:** Hi-C metagenomics, avian gut microbiome, antimicrobial resistance genes, mobile genetic elements, aquaculture, One Health

## Abstract

The double-crested cormorant (*Nannopterum auritum*), a piscivorous bird endemic to North America, frequently forages in aquaculture ponds during migration and wintering, contributing to economic losses in catfish-producing regions of the southern United States. While interactions between cormorants and aquaculture systems are well documented, their associated microbial communities and genetic elements remain less characterized. In this exploratory study, Hi-C-enabled metagenomics was applied to fecal samples from two cormorants to generate a genome-resolved, descriptive analysis of gut microbial composition and to associate bacterial genomes with mobile genetic elements (MGEs), antimicrobial resistance genes (ARGs), and putative virulence-associated genes. Metagenome-assembled genomes (MAGs) included taxa reported in aquatic or animal-associated environments, including *Edwardsiella tarda*, *Plesiomonas shigelloides*, *Clostridium perfringens*, and *Campylobacter volucris*. ARGs were detected across multiple MAGs, with *E. tarda* harboring the greatest diversity. Hi-C-enabled linkage of plasmids and phages to putative hosts, providing structural insight into microbial organization. Analyses are descriptive (*n* = 2) and do not include statistical comparisons or diversity metrics. These findings demonstrate the utility of Hi-C for resolving gene–host associations and provide a framework for future studies of microbial connectivity in One Health contexts.

## 1. Introduction

Aquaculture is an integral component of the global food system, contributing to sustainable food security and providing a critical source of protein for a growing population [1,2]. In the United States, the aquaculture sector generates approximately $1.5 billion annually, with catfish accounting for 74% of finfish production as of 2019 [3,4]. Production is concentrated in the Southern United States, where Mississippi leads the industry, contributing 54% of the nationwide market-size channel catfish (*Ictalurus punctatus*) and hybrid catfish (channel catfish female × blue catfish male, *Ictalurus furcatus*) sales [5,6]. Beyond its contribution to food production, this industry plays a vital economic role by supporting employment and income in rural communities [7,8]. Given its economic importance at both regional and national levels, maintaining fish health and production stability is critical for food security and industry sustainability.

Despite its importance, catfish aquaculture faces significant challenges, with disease representing a major threat to production efficiency and profitability. Bacterial pathogens, including *Edwardsiella* spp., virulent *Aeromonas hydrophila*, and *Flavobacterium covae*, are among the leading causes of morbidity and mortality, resulting in economic losses due to fish death, reduced growth, and increased reliance on treatments and vaccines [9,10,11]. In many cases, these infections are opportunistic, occurring when fish are stressed by suboptimal conditions such as overstocking, poor water quality, or inadequate management practices [12].

Transmission of bacterial pathogens in aquaculture systems occurs through multiple routes, including direct fish-to-fish contact, ingestion of infected individuals, contaminated equipment, and environmental exposure [10]. Among these routes, interactions with wildlife, particularly piscivorous birds, represent an understudied but potentially important pathway. These birds frequently forage in aquaculture ponds, where they are primarily recognized for causing direct losses through predation [13]. However, they may also contribute to disease dynamics by introducing pathogens and parasites into aquatic systems, most commonly through fecal shedding [14,15,16]. For instance, piscivorous birds are known to play a key role in the life cycle of the trematode *Bulbophorus damnificus*, transmitting the parasite to catfish via fecal depositions into ponds [17,18].

Although several studies have investigated the role of fish-eating birds in pathogen dissemination, research specifically addressing bacterial transmission by double-crested cormorants is limited. One study by Cunningham et al. demonstrated that cormorants can viably shed a hypervirulent strain of *A. hydrophila* in their feces, suggesting their potential to act as vectors for additional bacterial pathogens [16]. More broadly, wild birds have been recognized as reservoirs of diverse microbial communities, including bacteria associated with antimicrobial resistance (AMR), with implications for animal, environmental, and public health [19,20,21]. Despite this, the extent to which piscivorous birds harbor bacteria, antimicrobial resistance genes (ARGs), and mobile genetic elements (MGEs) relevant to aquaculture systems remains poorly defined, representing a key knowledge gap at the wildlife–environment–aquaculture interface.

Bacterial pathogens are highly dynamic, evolving through genetic mutations and horizontal gene transfer (HGT), often mediated by MGEs such as plasmids and bacteriophages [22,23,24]. These processes facilitate the acquisition of ARGs and virulence factors, which can reduce treatment efficacy and contribute to therapeutic failure in aquaculture settings [25,26,27]. Virulence factors, including adhesions, toxins, and secretion systems, enable bacterial invasion and persistence within hosts, while MGEs promote the rapid dissemination of these traits across populations and environments [28,29,30,31]. Interspecies gene transfer further expands pathogenic potential by enabling bacteria to acquire novel functional capabilities [32,33].

Understanding the distribution and linkage of these genetic elements within complex microbial communities requires advanced analytical approaches. Traditional sequencing methods, such as 16S rRNA amplicon sequencing and short-read shotgun sequencing, provide valuable taxonomic and functional insights but are limited in their ability to resolve genome structure and associate MGEs with specific hosts. Hi-C sequencing, a proximity ligation technique derived from Chromosome Conformation Capture (3C), overcomes these limitations by capturing the three-dimensional organization of DNA within cells [34,35,36]. By linking physically proximal DNA fragments, Hi-C enables improved genome assembly and binning, resolution of closely related strains, and direct association of plasmids and phages with their bacterial hosts [37,38,39,40]. This capability is particularly advantageous for analyzing complex metagenomes, where distinguishing individual genomes and their associated elements is critical.

Therefore, the objective of this study was to apply Hi-C-enabled metagenomics to characterize the gut microbiome of double-crested cormorants and to resolve associations between bacterial genomes, mobile genetic elements, virulence-associated genes, and AMR genes. Using fecal samples from two individuals, this study provides a genome-resolved, descriptive analysis of microbial community structure and genetic linkages. This work serves as proof of concept for applying Hi-C approaches to wildlife-associated microbiomes. It establishes a framework for future studies investigating microbial and genetic connectivity at the aquaculture–wildlife interface.

## 2. Materials and Methods

### 2.1. Collection of Double-Crested Cormorant Fecal Samples

Double-crested cormorants that migrate to the southern United States in the winter typically arrive in Mississippi in October–November and depart for northern breeding grounds in spring (March–May). During the 2021 fall field sampling season of the National Wildlife Research Center-Mississippi Field Station, fecal samples from two healthy individual double-crested cormorants (ID: 13009 and 48971) were collected from night roosts along the Tennessee-Tombigbee waterway near catfish farms located in east Mississippi, USA, as part of an ongoing study (QA 3039).

All bird capture, handling, and transmitter attachment procedures were conducted in accordance with federal wildlife permits and approved animal care protocols under the National Wildlife Research Center (NWRC) Institutional Animal Care and Use Committee (QA 1969, 2040, 2105, 3039).

Two fecal samples were collected from each cormorant, one for sequencing and the other retained as a backup. The birds were humanely captured and restrained during transmitter attachment. Each cormorant had a Cellular GPS (Evolution Series 400 GPS transmitter plus accelerometer from Cellular Tracking Technologies, Rio Grande, NJ, USA). These transmitters (not to exceed 3% total body weight) were placed on each cormorant using a modified harness design [41]. Transmitters were affixed dorsally between the wings using two Teflon ribbon loops positioned around the body, one anterior (neck loop) and one posterior (body loop) to the wings. Both loops were connected ventrally by a short loop of ribbon positioned over the sternum. Loops were secured using Loctite 380 glue and metal ferrules covered by shrink tubing [42].

### 2.2. Library Preparation and Sequencing

**Figure 1:**
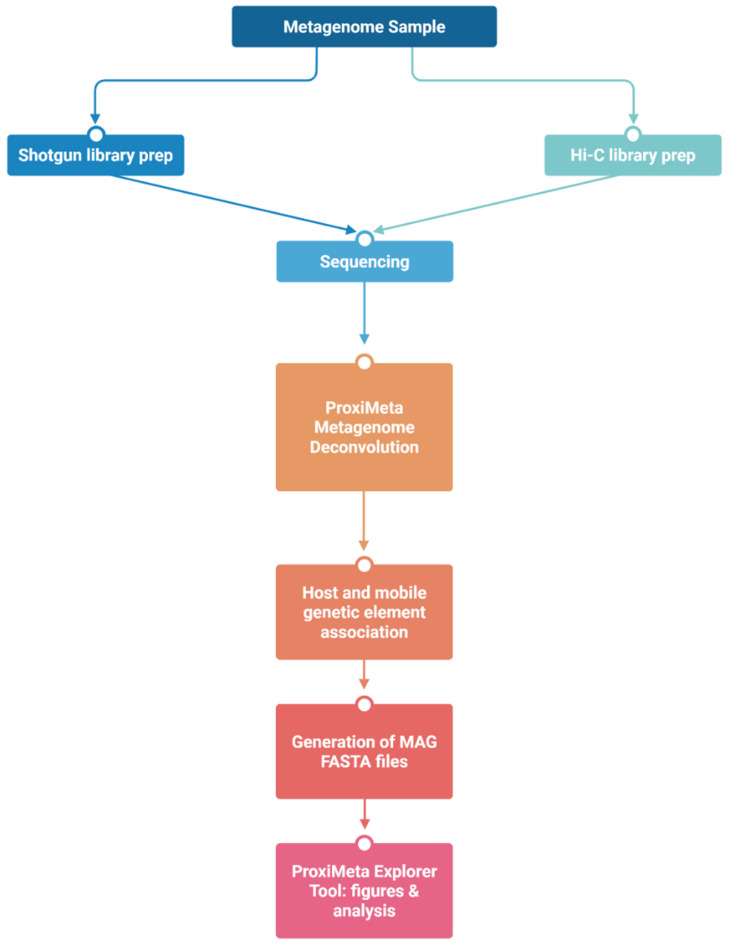
A flowchart depicting the ProxiMeta Hi-C workflow from library preparation to metagenome deconvolution and subsequent analysis through the Phase Genomics cloud-based bioinformatics portal (i.e., ProxiMeta Explorer). Created in BioRender. Sydney O’Donald. (2025) https://BioRender.com/y5pxtqx.

Fecal samples were shipped on dry ice via overnight delivery to Phase Genomics in Seattle, Washington, USA [43] for library preparation and Hi-C sequencing (Figure 1). The Phase Genomics ProxiMeta Hi-C v4.0 Kit was used to build the Hi-C library following the manufacturer’s protocol (Phase Genomics, Seattle, WA, USA) [39,44,45,46,47]. Briefly, intact cells were crosslinked through a formaldehyde solution, digested via Sau3AI and MluCI restriction enzymes, and proximity ligated with biotinylated nucleotides, producing chimeric molecules that comprise fragments from different regions of genomes that were physically close in proximity in vivo. Streptavidin beads were then used to pull down the proximity-ligated DNA molecules, followed by processing into an Illumina-compatible sequencing library. Separately, aliquots of the samples were processed for shotgun metagenomic sequencing by extracting DNA using a ZYMObiomics DNA miniprep kit (Zymo Research Corp., Irvine, CA, USA) to create a metagenome shotgun library using ProxiMeta library preparation reagents. Sequencing was performed via the Illumina NovaSeq platform (Illumina, Inc., San Diego, CA, USA), generating PE150 read pairs for both Hi-C and shotgun libraries.

For each DNA extraction, a long-read sequencing library was also prepared using the Ligation Sequencing kit 1D (SQK-LSK109; Oxford Nanopore Technologies, Oxford, UK) and sequenced on a FLO-MIN106 (R9.4.1) MinION flow cell (Oxford Nanopore Technologies, Oxford, UK). Because the cormorant fecal samples represented high-complexity microbial communities, the Hi-C libraries comprised 50 million pairs (2 × 75 base pairs or longer), and the shotgun libraries from the same samples were based on reads from 200 million pairs (2 × 75 base pairs or longer). The Hi-C and shotgun metagenomic sequencing files were then uploaded to the Phase Genomics cloud-based bioinformatics portal for further analysis.

### 2.3. Metagenome Assembly, Quality Assessment, and Taxonomic and Functional Assignment of Assembled Microbes

The shotgun libraries were filtered, trimmed for quality, and normalized using fastp v0.20.1 [48]. The libraries were assembled using MEGAHIT v1.2.9 [49] using default options. Hi-C reads were then aligned to the assembly using the Hi-C kit manufacturer’s recommendations. Reads were aligned using BWA-MEM v.0.7.17 [50] with the specified-5SP option and all other options default. PCR duplicates and invalid Hi-C read pairs from preprocessing were removed using proprietary tools from the cloud platform. Metagenome deconvolution and binning of metagenome-assembled genomes (MAGs) was performed via the ProxiMeta platform [44]. The resulting putative genome and genome fragment clusters were assessed for quality via CheckM v.1.1.3 [51] and assigned taxonomic classifications through GTDB-Tk v2.1.0 [52].

MAGs were classified as “known” or “novel” using the Phase Genomics ProxiMeta pipeline. Genome quality was assessed by completeness (>70%) and marker gene overrepresentation (MGO < 10%), where MGO reflects duplicated single-copy marker genes and serves as a proxy for contamination. The ProxiMeta novelty score is based on sequence similarity to reference genomes (e.g., average nucleotide identity and gene content). MAGs with scores < 90% were classified as “known”, whereas those >90% were classified as “novel”, following default ProxiMeta criteria. These classifications are relative to current database representation. These default thresholds were used in further analyses to view more comprehensive results of each metagenome and its components. While increasing the completeness threshold to 90% would give a more conservative and confident analysis of MAGs, it limits the scope by selecting the few, near-complete genomes. Additionally, this approach would reduce the number of host genomes available for associating mobile genetic elements (MGEs). In this instance, lower thresholds were kept to assess broad community diversity and ecological breadth within the samples.

MAGs were annotated using the Rapid Annotation using Subsystem Technology platform (accessed 2024) [53] to characterize the features of each genome. These annotated genomes were subsequently used in downstream analyses. The efficacy of both platforms was validated because the annotations from each MAG shared similar results for bacterial identification. For microbe annotation, the following criteria were chosen for the annotation pipeline: classic RAST for annotation, RAST gene caller for open reading frame (ORF) identification, and FIGfam (version release 70 with automatic fix errors and fix frameshifts options). Virulence factors encoded within the assembled and annotated genomes were identified by searching against the Virulence Factor Database 2025 [54]. Nucleotide files were blasted against the virulence factor database using CLC Genomics Workbench (version 24). Results were processed into a presence/absence matrix via Python v3.11 and visualized as a heatmap in RStudio v2024.04.2 using default options. An E-value < 1 × 10^−150^ was used as the threshold value. The resulting output data was visualized as a heatmap, which was created using the default options of the R library heatmap.

Antimicrobial resistance genes (ARGs) were annotated using NCBI’s AMRFinderPlus v.3.10.5 [55] with the ‘—plus’ option specified and all other options default. Additionally, ARGs linked to specific genomes were identified through the Comprehensive Antibiotic Resistance Database (CARD) v3.2.4 [56]. Nucleotide files of sequenced genomes were submitted to perform a BLASTN search through NCBI BLAST+ v2.16.0, and the presence and absence of the AMR genes were recorded.

### 2.4. Mobile Genetic Elements

Mobile genetic element (phage and plasmid) contigs were identified from the MAGs of each sample using proprietary identification pipelines. Phage elements were identified using VIBRANT v1.2.1 [57], and the percentage of viral DNA represented in assembly was assessed through CheckV 1.0.3 [58]. Additionally, the Phage Search Tool with Enhanced Sequence Translation (PHASTEST; accessed 2024) [59] was used to assess the number of phage elements within each genome. The bacterial annotation mode was set to Lite, utilizing the SwissProt database. The calculated results were arranged into three categories: a score > 90 was considered an intact phage element, a score between 70–90 was deemed questionable, and a score < 70 was considered incomplete. Plasmid completeness was determined based on the percentage of coverage aligned to the best hit reference sequence from the NCBI RefSeq plasmid database (accessed 2022) [60]. Host taxonomy assignments from GTDB-Tk were used for all phage–host, plasmid–host, and AMR–host connection-related analyses.

### 2.5. Abundance Analysis

Abundance calculations for microbes, phages, and plasmids were calculated through the ProxiMeta Explorer tool v.0.5.0.1 [46]. Forward shotgun reads were mapped to each corresponding assembly using bowtie2 v.2.5.4. Transcripts per kilobase million (TPM) values were calculated for each MAG (bacterial, plasmid, and phage) as the normalized abundance metric representing the transcripts per kilobase of the genome length per million transcripts. This metric accounts for both the length of the genome and the depth of the sequencing and was subsequently used to represent the data.

## 3. Results

### 3.1. Microbial Composition of Metagenome Samples

Fecal samples 13009 and 48971 will be referred to as ‘Bird A’ and ‘Bird B’ moving forward. ProxiMeta assemblies for both samples (Table 1, Table 2, Tables S1 and S2) included metagenome-assembled genomes classified as “known” or “novel” based on completeness, contamination (MGO), and sequence similarity to reference genomes as defined by the ProxiMeta pipeline (see Methods Section 2.3).

Bird A produced a total assembly length of 993,740,580 bp across 399,340 contigs, organized into 162 clusters (Table 1 and Table S1). Of these, 22 MAGs met the >70% completeness threshold (22/162; 13.6%), including 15 with >90% completeness and 9 with >95% completeness. Among these 22 MAGs, 6 were classified as known and 16 as novel.

Bird B produced a total assembly length of 1,338,254,414 bp across 120,972 contigs, organized into 66 clusters (Table 2 and Table S2). Of these, 25 MAGs met the >70% completeness threshold (25/66; 37.9%), including 16 with >90% completeness and 7 with >95% completeness. Among these 25 MAGs, 2 were classified as known and 23 as novel.

**Figure 2:**
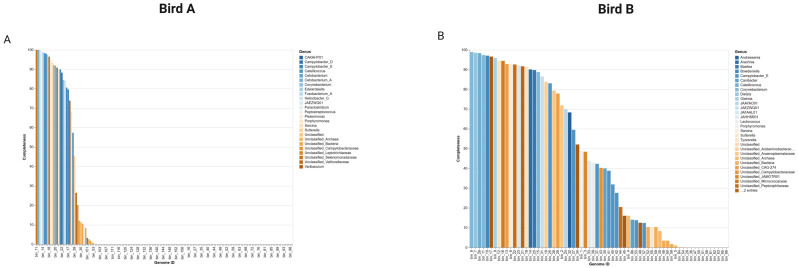
Analysis of microbial genome bins and completeness within Bird A and Bird B. Unique bacterial isolates at a genus taxonomic level are coordinated by color. (**A**) Bin overview of each isolate and its completeness after Hi-C sequencing in Bird A. (**B**) Bin overview of each isolate and its completeness after Hi-C sequencing in Bird B.

**Figure 3:**
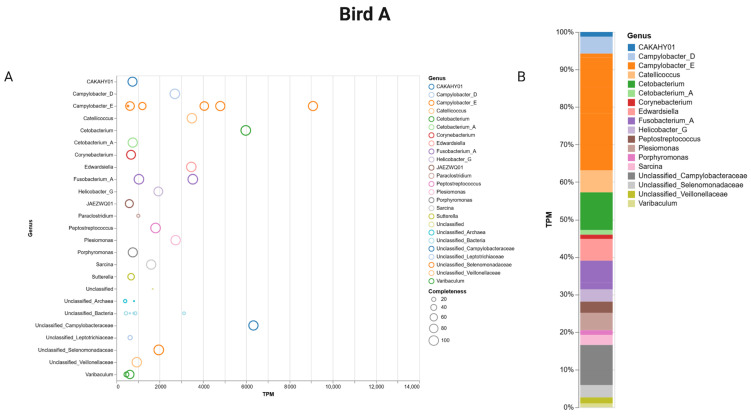
Analysis of microbes in Bird A. Unique bacterial isolates at a genus taxonomic level are coordinated by color. (**A**) Breakdown of microbe presence by genus and transcripts per kilobase million (TPM), with ring size indicating the level of completeness. (**B**) Percentage of abundance of isolates within the metagenome by genus and TPM. To be noted, *Sarcina* represents *Clostridium perfringens* within the data.

**Figure 4:**
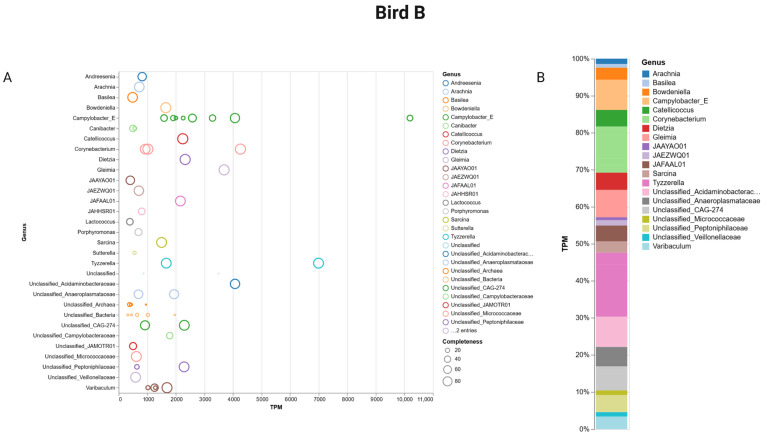
Analysis of microbes in Bird B. Unique bacterial isolates at a genus taxonomic level are coordinated by color. (**A**) Breakdown of microbe presence by genus and transcripts per kilobase million (TPM), with ring size indicating the level of completeness. (**B**) Percentage of abundance of isolates within the metagenome by genus and TPM. To be noted, *Sarcina* represents *Clostridium perfringens* within the data.

Across both samples, reconstructed prokaryotic genome sizes ranged from approximately 0.7 to 4.0 Mb. The completion percentage of MAGs within each sample followed a positive skew (Figure 2A,B and Figure S1A,D), highlighting the accuracy of the Hi-C sequencing and classification of unique microbes identified within Bird A and Bird B (Figure 3 and Figure 4).

### 3.2. Mobile Genetic Element Composition of Metagenome Samples

**Figure 5:**
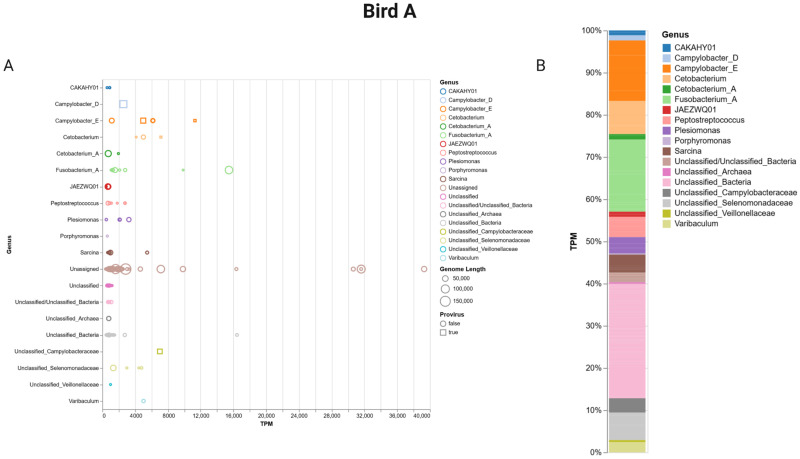
Analysis of viral elements in Bird A. Unique bacterial isolates at a genus taxonomic level are coordinated by color. (**A**) Breakdown of virus–host association by genus and transcripts per kilobase million (TPM), with ring size indicating viral genome size (bp) and a square representing a provirus. (**B**) Percentage of abundance of viral elements within the fecal sample by host genus and TPM. To be noted, *Sarcina* represents *Clostridium perfringens* within the data.

**Figure 6:**
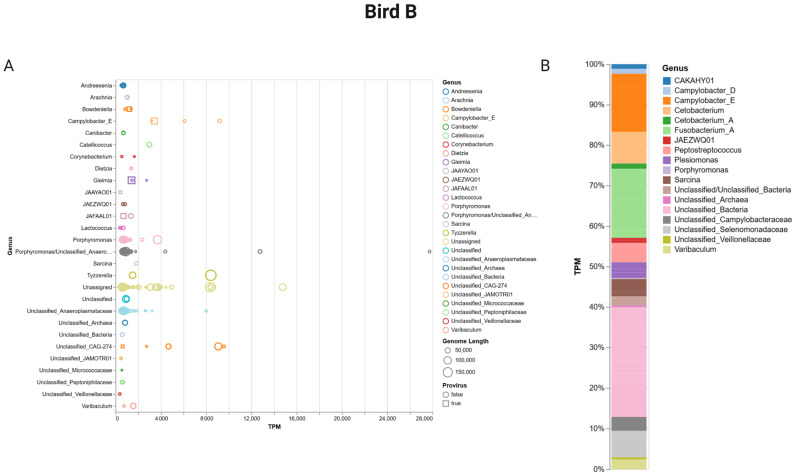
Analysis of viral elements in Bird B. Unique bacterial isolates at a genus taxonomic level are coordinated by color. (**A**) Breakdown of virus–host association by genus and transcripts per kilobase million (TPM), with ring size indicating viral genome size (bp) and a square representing a provirus. (**B**) Percentage of abundance of viral elements within the fecal sample by host genus and TPM. To be noted, *Sarcina* represents *Clostridium perfringens* within the data.

Hi-C proximity ligation identified associations between viral elements and putative bacterial hosts in both samples (Figure 5, Figure 6 and Figure S2A,C).

In Bird A (Figure 5 and Figure S2B), phage associations were most frequently observed in taxa classified within *Campylobacter* and *Fusobacterium.* Prophage elements were detected in members of the families Campylobacteraceae and Fusobacteriaceae. More than half of the detected viral elements (>50%) were not assigned to a host genome.

In Bird B (Figure 6 and Figure S2D), viral associations were concentrated in unclassified taxa within the family Anaeroplasmataceae and the genus *Porphyromonas.* As in Bird A, a majority of viral elements (>50%) were not linked to a host genome.

PHASTEST analysis (Table S3) identified intact prophage elements in five MAGs: *Clostridium perfringens* ATCC 13124, *E. tarda* ATCC 15947, *Clostridiales* (UID1375), *Epsilonproteobacteria* (UID3066), and *Catellicoccus marimammalium* M35/04/03. Two MAGs (*Clostridiales*, UID 1212) contained questionable prophage elements. Incomplete prophage elements were identified in *Actinomycetales* (UID 1531), *Clostridiales* (UID 1212), and *E. tarda* ATCC 15947.

**Figure 7:**
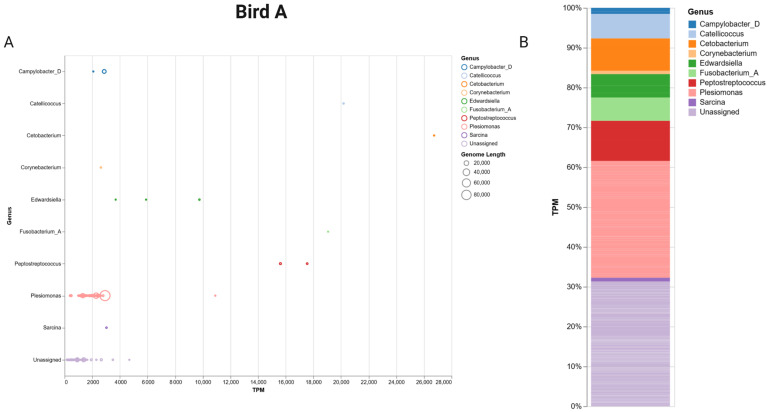
Analysis of plasmids in Bird A. Unique bacterial isolates at a genus taxonomic level are coordinated by color. (**A**) Breakdown of plasmid-host association by genus and transcripts per kilobase million (TPM), with ring size indicating plasmid size (bp). (**B**) Percentage of abundance of plasmids within the fecal sample by host genus and TPM. To be noted, *Sarcina* represents *Clostridium perfringens* within the data.

**Figure 8:**
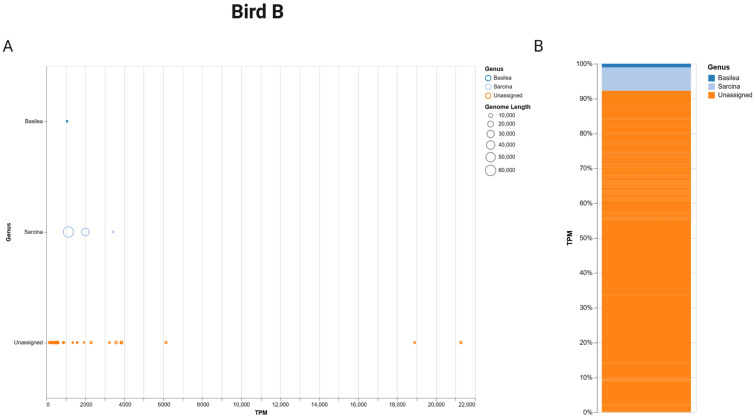
Analysis of plasmids in Bird B. Unique bacterial isolates at a genus taxonomic level are coordinated by color. (**A**) Breakdown of plasmid-host association by genus and transcripts per kilobase million (TPM), with ring size indicating plasmid size (bp). (**B**) Percentage of abundance of plasmids within the fecal sample by host genus and TPM. To be noted, *Sarcina* represents *Clostridium perfringens* within the data.

Plasmid sequences were also identified and linked to putative hosts using Hi-C (Figure 7, Figure 8 and Figure S3A,C). In Bird A (Figure 7 and Figure S3B), plasmid associations were most frequently observed in *Plesiomonas shigelloides*. Additional plasmids were linked to *E. tarda* (*n* = 3), a *Campylobacter* MAG (*n* = 2), and *C. marimammalium* (*n* = 1). One plasmid associated with *P. shigelloides* was 90,232 bp in length. A substantial portion of plasmids in this sample were not assigned to a host.

In Bird B (Figure 8 and Figure S3D), plasmids were associated with two genera: *Clostridium* and *Basilea*. The largest plasmids (~30 kb and ~60 kb) were linked to *C. perfringens*. More than 90% of plasmid sequences in Bird B were not assigned to a host genome.

### 3.3. Linking Mobile Elements to Host Microbes Within the Metagenome

**Figure 9:**
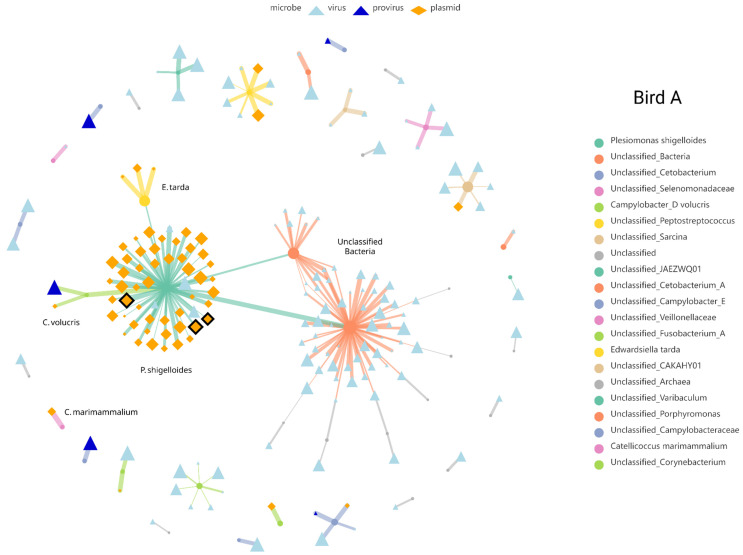
Linkage map of phages, plasmids, and AMR genes to their host microbes in Bird A. The map was set to the species taxonomic rank setting, adjusted inter-linkage vs. intra-linkage ratio edge metric setting, with gene resistance showing. Microbes (circle with color), viruses (light blue triangle), proviruses (dark blue triangle), and plasmids (orange diamond; black outline if carrying ARGs) are shown in each sample. Interactions via proximity ligation are represented by linear connections, with bacterial isolates determined by color. The thickness of the lines determines the confidence of the interaction, with a thick line indicating strong confidence in the sharing of genetic material. If a genetic element, such as a plasmid, is connected by the lines of two different microbes, it indicates HGT. The names of known bacterial species within the sample are listed next to their representation marker on the map. Additionally, bacterium names are listed on the map if they are contributing to any exchange of genetic material. To be noted, *Sarcina* represents *Clostridium perfringens* within the data.

**Figure 10:**
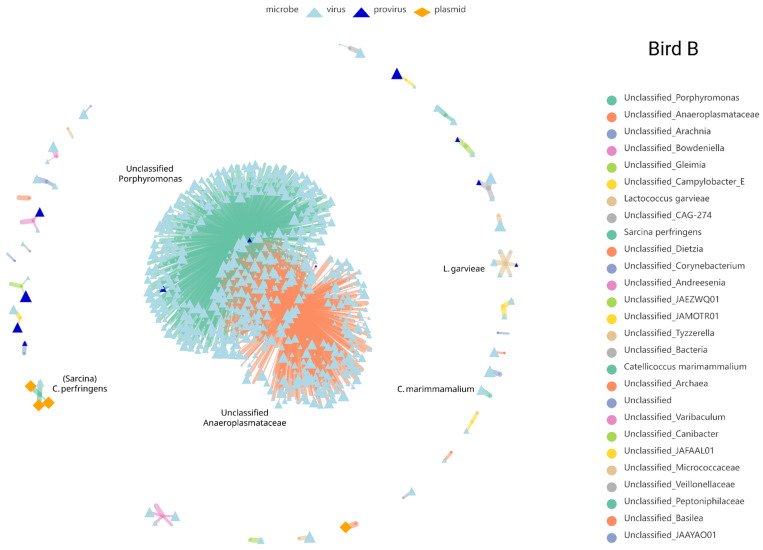
Linkage map of phages, plasmids, and AMR genes to their host microbes in Bird B. The map was set to the species taxonomic rank setting, adjusted inter-linkage vs. intra-linkage ratio edge metric setting, with gene resistance showing. Microbes (circle with color), viruses (light blue triangle), proviruses (dark blue triangle), and plasmids (orange diamond; black outline if carrying ARGs) are shown in each sample. Interactions via proximity ligation are represented by linear connections, with bacterial isolates determined by color. The thickness of the lines determines the confidence of the interaction, with a thick line indicating strong confidence in the sharing of genetic material. If a genetic element, such as a plasmid, is connected by the lines of two different microbes, it indicates HGT. The names of known bacterial species within the sample are listed next to their representation marker on the map. Additionally, bacterium names are listed on the map if they are contributing to any exchange of genetic material. To be noted, *Sarcina* represents *Clostridium perfringens* within the data.

Integration of Hi-C linkages, MAG reconstruction, and GTDB-Tk taxonomy enabled identification of associations between bacterial genomes, MGEs, and ARGs (Figure 9 and Figure 10).

In Bird A (Figure 9), *P. shigelloides* showed the highest number of MGE associations, including >50 linked plasmids of varying sizes. Three plasmids associated with this genome contained ARGs (*floR*, *tetA*, *sul2*). Hi-C linkages also connected *P. shigelloides* with *E. tarda*, *C. volucris*, and two unclassified taxa. Other MAGs showed fewer associations, primarily limited to plasmids or viral elements.

In Bird B (Figure 10), fewer MGE-host associations were detected overall. The highest number of viral linkages was observed in unclassified Anaeroplasmataceae and *Porphyromonas.* Other taxa showed limited associations with plasmids or phages, and inter-bacterial linkages were not observed.

### 3.4. Virulence Genes Associated with MAGs

**Figure 11:**
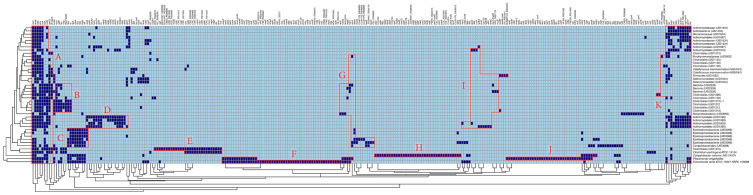
Heatmap of combined MAGs with greater than 70% completeness from Bird A and Bird B showing putative virulence genes. Putative virulence genes are listed at the top of the heatmap. Unique microbes are listed to the right of the heatmap. Genes present in a bacterial isolate’s genome are indicated by dark blue squares. (**A**) Cluster of virulence genes that encode a range of virulence factors, including adhesion, motility, and invasion. (**B**) A small cluster confined to the *Clostridiales* order with genes for adhesion and flagella. (**C**) A small cluster of flagellar genes under *Epsilonproteobacteria*. (**D**) A small cluster of genes under *Actinomycetales* covering various functions. (**E**) A cluster of virulence genes shared by *C. perfringens* ATCC 13124 and *Clostridiales* (UID1375) encoding virulence factors common to *Clostridium* spp. (**F**) A cluster of virulence genes under *E. tarda* ATCC 15947 NBRC 105688 encoding several virulence factors of different functions with origins from an array of bacterial species. (**G**) Scattered virulence genes with very little correlation. (**H**) A cluster of virulence genes in *C. volucris* LMG 24379 that are common to *Campylobacter* spp. (**I**) Scattered genes with very little correlation. (**J**) A cluster of virulence genes in *P. shigelloides* that encode secretion system maintenance and function. (**K**) Virulence gene clusters that are commonly linked to *Mycobacterium* spp.

Putative virulence-associated genes were identified in MAGs with >70% completeness and are summarized in Figure 11. Gene presence patterns were grouped into clusters based on co-occurrence.

Clusters A and K included genes detected across the majority of MAGs. Clusters E, F, H, and J contained genes restricted to specific MAGs, including those similar to *C. perfringens* ATCC 13124 or *E. tarda* ATCC 15947, for example. Clusters B, C, and D contained genes shared among subsets of MAGs. Clusters G and I did not show consistent distribution patterns across taxa.

### 3.5. Antimicrobial Resistance Genes Associated with MAGs

ARGs were identified in MAGs with >70% completeness (Table 3). ARGs were detected in the majority of genomes across both samples.

Bird A contained ARGs spanning multiple antibiotic classes. In contrast, ARGs detected in Bird B were primarily associated with glycopeptide resistance. *E. tarda* contained the highest number of ARGs and the broadest range of antibiotic classes among all MAGs.

ARGs were identified in multiple taxa across both samples, including genomes previously associated with animal or environmental microbiomes.

## 4. Discussion

**Figure 12:**
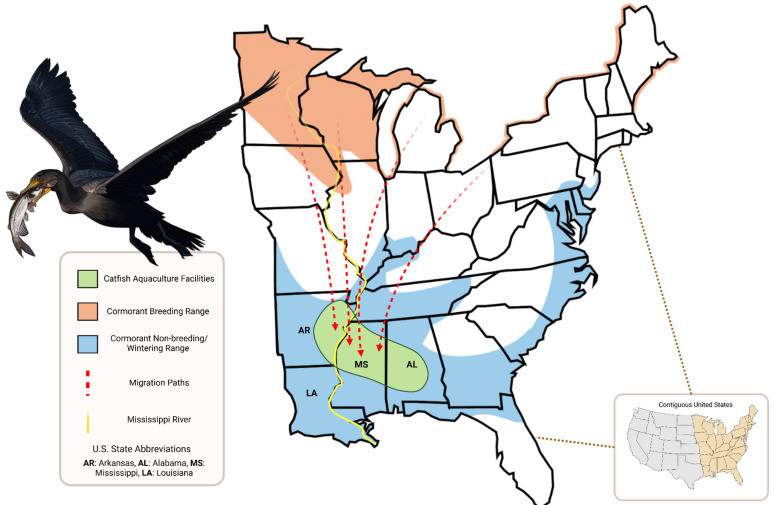
A map depicting double-crested cormorant migration patterns in the eastern United States. Orange-highlighted regions represent ranges of habitat occupied by double-crested cormorants during the breeding season. Blue-highlighted areas represent ranges of habitat occupied by cormorants during the wintering/non-breeding season. The red arrows indicate the migration route of cormorants from their breeding grounds to their common wintering areas. A large number of cormorants migrate along the Mississippi River, shown in yellow, and situate themselves near catfish aquaculture ponds as they provide an easy source of food. The green highlighted area encompasses the region where the majority of catfish aquaculture facilities are established within the United States. For the small USA inset map, the tan color highlights the eastern United States region shown in detail in the main map, while the gray states represent the rest of the contiguous United States outside the focus area. Created in BioRender. Sydney O’Donald. (2025) https://BioRender.com/x890i8q.

Predation from piscivorous birds is a well-documented challenge for catfish aquaculture in the United States. Double-crested cormorants (*Nannopterum auritum*; formerly *Phalacrocorax auritus*) are frequently observed foraging in aquaculture ponds and have been associated with substantial economic losses in catfish-producing regions [61,62,63,64]. This species is widely distributed across North America, breeding in northern regions and migrating southward during wintering periods (Figure 12) [65]. In the Mississippi Delta, overlap with the Mississippi Flyway provides frequent opportunities for interaction with aquaculture systems [62,63,64,66].

While ecological and economic impacts of cormorant foraging behavior are well established, less is known about the composition and genetic content of their gut-associated microbiota in relation to aquaculture environments. In this study, Hi-C-enabled metagenomics was used to generate a genome-resolved, descriptive characterization of fecal microbiomes from two individual birds. This analysis focused on MAG recovery (>70% completeness), taxonomic assignment, and the identification of MGEs, ARGs, and putative virulence-associated genes. All analyses are descriptive (*n* = 2) and do not include statistical comparisons, diversity metrics, or quantitative assessments of between-sample differences.

Recovered MAGs included taxa commonly reported in avian and other host-associated gut microbiomes, including members of *Clostridiales*, *Campylobacterales*, *Fusobacteriales*, *Lachnospirales*, and *Actinomycetales* [67]. *Campylobacter* spp. was among the most abundant taxa in both samples (Figure 3, Figure 4 and Figure S1B,C,E,F), consistent with their known presence in wild bird microbiomes [68]. These observations align with previous reports of avian-associated bacterial communities, although the present study does not evaluate prevalence or ecological distribution beyond the sampled individuals.

One MAG identified in Bird B, classified as *Tyzzerella*, has been previously reported in human gut microbiome studies with variable prevalence linked to host-related factors [69,70,71,72]. Its detection in this dataset may reflect environmental exposure pathways, including anthropogenic inputs; however, no source attribution can be inferred from the present data.

Differences in MAG composition between Bird A and Bird B were observed, including variation in the proportion of known versus novel classification. These differences may reflect individual-level biological variation and/or methodological factors such as sequencing depth, assembly complexity, and Hi-C binning performance (Figure 3 and Figure 4). Given the limited sample size, these observations are not evaluated statistically.

Several MAGs identified as “known” included taxa previously reported in aquaculture or animal-associated contexts, such as *E. tarda* and *P. shigelloides*. *Edwardsiella* spp. are well-documented pathogens in catfish aquaculture systems [73,74], while *P. shigelloides* has been reported in environmental and animal-associated contexts and has been associated with ARG carriage [75,76]. Other detected taxa, including *C. volucris*, *C. marimammalium*, and *Clostridium* spp., have been associated with enteropathogenesis or bacteremia in humans and animals [77,78,79]. Their detection reflects taxonomic presence within the samples and does not imply pathogenic activity.

MGEs were identified through Hi-C linkage and reference-based validation, including plasmids and prophage elements. Viral associations were observed across multiple taxa in both samples, with a substantial proportion of viral elements not assigned to a host genome. This is consistent with known limitations in viral host assignment in metagenomic datasets [80]. Similarly, plasmid-host linkages were unevenly distributed, with a large proportion of plasmids remaining unassigned, reflecting possible limitations in reference databases and assembly resolution [81]. However, these plasmids represent a floating gene pool within the metagenome, accessible to different bacteria through HGT.

In Bird A, *P. shigelloides* showed the highest number of plasmid associations, including plasmids carrying ARGs (*floR*, *tetA*, *sul2*). In Bird B, plasmid associations were limited to a smaller number of taxa, with *C. perfringens* carrying the largest plasmids identified in that sample. These observations describe the distribution of MGEs within each dataset and do not infer transfer dynamics.

Putative virulence-associated genes were detected across multiple MAGs and grouped into shared and taxa-associated clusters (Figure 11). Broadly distributed gene clusters included functions related to stress response and cellular maintenance, which are widely conserved across bacterial taxa [82,83,84]. Taxon-associated clusters reflected functions previously described in corresponding bacterial groups, including survival and invasion mechanisms such as secretion systems, motility, and nutrient acquisition pathways [85,86,87,88,89,90,91,92,93,94,95,96,97,98,99,100,101]. The presence of these genes indicates functional potential based on annotation but does not reflect expression or pathogenic activity.

Virulence-associated gene annotations are based on sequence homology to curated databases and therefore represent putative functions. Due to conserved protein domains, incomplete MAG reconstruction, and limitations in reference databases, these annotations may include false positives and should be interpreted as potential functional signals rather than confirmed virulence determinants.

Similarly, ARG annotations are based on sequence similarity to curated resistance databases and therefore represent predicted resistance potential rather than confirmed phenotypic resistance. As with virulence gene annotation, false positives may arise due to conserved protein domains, fragmented MAG recovery, and incomplete reference representation, particularly for environmental taxa.

ARGs were identified across multiple MAGs in both samples (Table 3). *E. tarda* contained the highest diversity of ARGs across both datasets. The ARGs included were associated with efflux systems and antibiotic modification pathways previously reported in clinically relevant bacteria [54,102,103,104,105,106,107,108]. Gram-positive MAGs contained glycopeptide-associated resistance determinants, including genes linked to vancomycin resistance operons and peptide resistance mechanisms [54,109,110,111,112].

Several detected taxa are also relevant in the context of aquaculture-associated microbial ecology and antimicrobial resistance. While only two detected taxa, *Edwardsiella tarda* and *Plesiomonas shigelloides*, have previously been associated with disease in catfish or other aquatic organisms, both are relevant from an aquaculture and One Health perspective due to prior reports of zoonotic infections and antimicrobial resistance [113,114,115]. *Edwardsiella tarda* ATCC 15947 was originally isolated from human feces, although related strains have also been identified from fish and aquatic environments through sequencing-based studies [116]. Likewise, *P. shigelloides* has been reported across multiple animal hosts and environmental settings, including opportunistic infections in humans and aquatic organisms [117].

The detected MAGs also contained ARGs and putative virulence-associated genes linked to diverse cellular functions, highlighting the genomic diversity represented within these fecal microbiomes. For example, *P. shigelloides* was associated with plasmid-linked ARGs, including *floR*, *tetA*, and *sul2*, while *E. tarda* contained multiple genes associated with multidrug resistance systems, including components of the *adeFGH* and *acrAB-tolC* efflux systems. These observations describe the genetic content associated with recovered MAGs and demonstrate the ability of Hi-C-enabled metagenomics to resolve potential host–mobile element relationships within complex microbial communities. However, the presence of these genes does not indicate phenotypic resistance, pathogenic activity, or active horizontal transfer within the sampled microbiomes.

Collectively, these results describe the genome-resolved microbial and genetic composition of two cormorant fecal microbiomes and demonstrate the capacity of Hi-C-enabled metagenomics to link MAGs with MGEs and ARGs at a structural level. Importantly, this study is exploratory in scope and does not assess transmission, persistence, or epidemiological relationships. The dataset is limited to two individuals and does not support statistical inference regarding microbial differences between samples.

This work provides a proof-of-concept demonstration of Hi-C metagenomics in avian gut microbiomes and illustrates how genome-resolved approaches can organize microbial, plasmid, and phage-associated signals within complex communities. While not designed to assess transmission, these data may be integrated with environmental, livestock, and wildlife datasets in future studies to support broader One Health investigations of microbial ecology at the wildlife–aquaculture interface. Expansion to larger sample sizes, additional host species, and environmental sampling will be necessary to contextualize these observations and evaluate their broader ecological relevance.

## Figures and Tables

**Table 1:** Phase Genomics ProxiMeta results of cormorant Bird A.

Bird A									
Cluster ID	Top Reference	Completeness (%)	MGO (%)	Novelty Score	Abundance	Contig N50	Genome Size	Number of Contigs	GC (%)
bin 11	Firmicutes (UID1022)	100	1.48	98.01	0.08	97,977	1,901,298	34	34.77
bin 6	Bacteria (UID2329)	100	0.16	97.23	1.09	27,226	2,586,251	64	28.36
bin 7	Bacteria (UID2329)	100	1.12	97.62	0.4	163,501	2,200,486	120	28.23
bin 12	Clostridiales (UID1120)	99.3	0	98.8	0.06	51,471	1,894,079	84	30.59
bin 14	Bacteria (UID2329)	98.88	3.42	97.59	0.27	21,191	1,823,829	173	28.2
bin 23	*Catellicoccus marimammalium* M35/04/3	98.34	0.74	18.44	0.13	1,023,821	1,367,748	15	33.7
bin 21	*Campylobacter volucris* LMG 24379	98.05	0	12.12	2.93	77,015	1,506,994	48	28.17
bin 3	*Edwardsiella tarda* ATCC 15947 NBRC 105688	97.51	0.36	0.32	1.51	782,853	3,840,500	33	56.75
bin 18	Selenomonadales (UID1024)	96.47	0.15	98.35	0.09	56,858	1,622,822	78	38.32
bin 5	*Plesiomonas shigelloides*	94.63	1.84	12.03	0.46	10,168	3,268,833	373	51.55
bin 4	Clostridiales (UID1375)	93.55	0.81	81.74	0.11	123,993	3,295,749	59	27.58
bin 8	Bacteria (UID2329)	92.13	2.81	85.65	0.26	6250	2,139,963	330	31.17
bin 20	Epsilonproteobacteria (UID3066)	91.79	2.75	98.7	2.89	50,597	1,514,045	69	29.86
bin 19	Epsilonproteobacteria (UID3066)	90.79	0.43	98.68	1.32	118,224	1,550,314	50	36.74
bin 10	Porphyromonadaceae (UID2622)	90.03	0.79	98.67	0.51	6232	2,017,534	257	33.74
bin 13	Epsilonproteobacteria (UID3066)	89.87	8.27	99.11	8.79	33,637	1,849,317	170	54.6
bin 22	Clostridiales (UID1226)	88.25	1.27	98.65	0.34	5139	1,406,458	183	28.65
bin 9	Actinomycetales (UID1590)	84.47	1.45	92.64	0.18	8432	2,120,299	355	74.34
bin 29	Campylobacterales (UID3068)	84.27	0.82	96.26	0.01	249,928	1,248,954	9	35.79
bin 28	Epsilonproteobacteria (UID3066)	80.56	2.77	99.5	0.17	4948	1,263,334	220	39.88
bin 17	Epsilonproteobacteria (UID3066)	79.66	3.4	99	1.71	15,913	1,686,941	297	54.1
Assembly length (bp)								993,740,580	
Number of contigs total								399,340	
Number of contigs in clusters								21,063	
Percent assembly length in clusters								16.73	
Percent contigs in clusters								5.27	
Total clusters								162	
Complete clusters (>95% complete, <10% MGO)								9	
Excellent clusters (>90% complete, <10% MGO)								15	
Good clusters (>70% complete, <10% MGO)								22	
Reasonable clusters (>50% complete, <10% MGO)								24	

Top references for MAGs sequenced with a completeness > 75%. Hi-C MAG assembly information for the fecal sample is included in the bottom table.

**Table 2:** Phase Genomics ProxiMeta results of cormorant Bird B (sample 48971).

Bird B									
Cluster ID	Top Reference	Completeness (%)	MGO (%)	Novelty Score	Abundance	Contig N50	Genome Size	Number of Contigs	GC (%)
bin 8	Actinomycetales (UID1590)	98.91	1.59	94.07	0.22	71,588	2,609,314	89	74.79
bin 7	Actinomycetales (UID1590)	98.53	1.08	99.19	0.18	467,041	2,666,558	28	60.04
bin 29	*Catellicoccus marimammalium* M35/04/3	98.34	0.74	8.66	0.01	1,015,554	1,301,184	6	33.82
bin 11	Actinomycetales (UID1809)	97.4	0.75	98.37	0.07	87,253	2,135,811	55	60.98
bin 10	Actinobacteria (UID1454)	97.08	2.5	99.18	0.11	22,620	2,209,776	128	61.43
bin 17	Actinomycetaceae (UID1531)	96.61	1.75	99.17	0.04	13,823	1,775,997	38	48.51
bin 9	Actinomycetaceae (UID1531)	95.93	7.03	99.17	0.23	97,809	2,432,383	182	60.53
bin 12	Clostridiales (UID1212)	94.64	0.02	98.74	0.57	248,518	2,055,247	116	25.79
bin 16	Micrococcaceae (UID1623)	94.41	1.87	98.73	0.03	16,276	1,869,592	71	57.03
bin 13	Clostridiales (UID1212)	92.84	0.36	98.29	0.05	26,283	2,026,667	52	24.32
bin 6	*Clostridium perfringens* ATCC 13124	92.74	0	18.16	0.02	411,685	2,773,671	28	27.91
bin 22	Clostridiales (UID1125)	92.6	0.2	99.14	0.06	11,250	1,556,095	54	46.34
bin 23	Clostridiales (UID1120)	92.08	0.71	99.13	0.06	21,711	1,549,620	88	56.41
bin 21	Selenomonadales (UID1024)	91.67	1.35	98.7	0.06	4283	1,559,231	136	38.22
bin 18	Clostridiales (UID1212)	91.5	0.23	98.69	0.17	121,991	1,721,280	65	27.44
bin 14	Actinomycetales (UID1697)	90.1	1.18	98.67	0.03	20,360	1,975,032	162	61.58
bin 20	Betaproteobacteria (UID3959)	89.78	0.33	98.23	0.04	15,239	1,593,082	142	41.51
bin 15	Actinomycetales (UID1812)	88.78	2.69	98.66	0.05	13,413	1,973,127	190	62.05
bin 31	Clostridiales (UID1212)	86.52	0.67	99.08	0.03	4390	1,152,148	100	28.85
bin 19	Clostridiales (UID1120)	83.81	2.48	98.58	0.05	91,195	1,691,311	57	23.38
bin 25	Epsilonproteobacteria (UID3066)	83.07	1.04	99.52	0.15	30,502	1,475,623	93	50.27
bin 38	Bacteria (UID2328)	79.33	0	99	0.01	96,073	748,216	19	26.56
bin 26	Clostridiales (UID1212)	77.85	2.68	97.96	0.04	18,116	1,461,644	223	25.2
Assembly length (bp):								1,338,254,414	
Number of contigs total:								120,972	
Number of contigs in clusters:								14,866	
Percent assembly length in clusters:								18.05	
Percent contigs in clusters:								12.29	
Total clusters:								66	
Complete clusters (>95% complete, <10% MGO):								7	
Excellent clusters (>90% complete, <10% MGO):								16	
Good clusters (>70% complete, <10% MGO):								25	
Reasonable clusters (>50% complete, <10% MGO):								28	

Top references for MAGs sequenced with a completeness greater than 75%. Hi-C MAG assembly information for the fecal sample is included in the bottom table.

**Table 3:** Identified antimicrobial resistance genes of sample MAGs.

Drug Class		Glycopeptides							Aminoglycosides	Fluoroquinolones			Macrolide Antibiotics			Disinfecting Agents and Antiseptics	Cephalosporins	Peptide Antibiotics	Elfamycin
Assigned MAGs		*vanW*	*mprF*	*vanY*	*vanH*	*Van* ligase	*vanG*	*vanT*	*rpsL*	*adeF*	*gyrB*	*rsmA*	*KpnH*	*KpnF*	*CRP*	*qacG*	*PBP3*	*mprF*	*EF-Tu*
**Bird B**	bin 6—*Clostridium perfringens* ATCC 13124	**X**	**X**	**X**														**X**	
	bin 7—Actinomycetales (UID1590)																		
	bin 8—Actinomycetales (UID1590)																		
	bin 9—Actinomycetaceae (UID1531)				**X**														
	bin 10—Actinobacteria (UID1454)	**X**																	
	bin 11—Actinomycetales (UID1809)	**X**																	
	bin 12—Clostridiales (UID1212)	**X**					**X**	**X**											
	bin 13—Clostridiales (UID1212)	**X**		**X**			**X**	**X**											
	bin 14—Actinomycetales (UID1697)	**X**							**X**										
	bin 15—Actinomycetales (UID1812)	**X**																	
	bin 16—Micrococcaceae (UID1623)			**X**															
	bin 17—Actinomycetaceae (UID1531)																		
	bin 18—Clostridiales (UID1212)	**X**					**X**	**X**											
	bin 19—Clostridiales (UID1120)			**X**															
	bin 20—Betaproteobacteria (UID3959)															**X**			
	bin 21—Selenomonadales (UID1024)	**X**						**X**											
	bin 22—Clostridiales (UID1125)			**X**				**X**											
	bin 23—Clostridiales (UID1120)	**X**						**X**											
	bin 24—Actinomycetales (UID1697)																		
	bin 25—Epsilonproteobacteria (UID3066)																		
	bin 26—Clostridiales (UID1212)	**X**					**X**	**X**											
	bin 29—*Catellicoccus marimammalium* M35/04/3			**X**				**X**											
	bin 31—Clostridiales (UID1212)							**X**											
	bin 38—Bacteria (UID2328)																		
**Bird A**	bin 1—Firmicutes (UID1022)																		
	bin 3—*Edwardsiella tarda* ATCC 15947 NBRC 105688									**X**		**X**	**X**	**X**	**X**	**X**	**X**		
	bin 4—Clostridiales (UID1375)	**X**		**X**	**X**			**X**			**X**							**X**	
	bin 5—*Plesiomonas shigelloides*											**X**			**X**		**X**		**X**
	bin 6—Bacteria (UID2329)																		
	bin 7—Bacteria (UID2329)							**X**											
	bin 8—Bacteria (UID2329)							**X**											
	bin 9—Actinomycetales (UID1590)																		
	bin 10—Porphyromonadaceae (UID2622)							**X**											
	bin 12—Clostridiales (UID1120)	**X**						**X**											
	bin 13—Epsilonproteobacteria (UID3066)																		
	bin 14—Bacteria (UID2329)				**X**			**X**											
	bin 17—Epsilonproteobacteria (UID3066)																		
	bin 18—Selenomonadales (UID1024)	**X**						**X**											
	bin 19—Epsilonproteobacteria (UID3066)																		
	bin 20—Epsilonproteobacteria (UID3066)																		
	bin 21—*Campylobacter volucris* LMG 24379																		
	bin 22—Clostridiales (UID1226)	**X**																	
	bin 23—*Catellicoccus marimammalium* M35/04/3			**X**				**X**											
	bin 24—Actinomycetaceae (UID1531)																		
	bin 28—Epsilonproteobacteria (UID3066)																		
	bin 29—Campylobacterales (UID3068)																		

MAGs represented in Bird A and Bird B have a completeness >70%, with known genomes bolded. Present antimicrobial resistance genes are represented by X, which correspond to a specific gene. Each gene is grouped under its targeted antibiotic class.

## Data Availability

The Hi-C reads for this study are available in the NCBI–Sequence Read Archive (SRA) database under the accession code PRJNA1302101.

## Supplementary Materials

The following supporting information can be downloaded at: https://www.mdpi.com/article/10.3390/microorganisms14061198/s1, Table S1. Phase Genomics Hi-C ProxiMeta results of cormorant fecal sample 13009 (Bird A). Results provide each identified known and novel species associated with their bin number. Completeness percentage is given as well as marker gene overrepresentation, novelty score, abundance, contig N50, genome size, number of contigs, and GC percentage of each isolate. Table S2. Phase Genomics Hi-C ProxiMeta results of cormorant fecal sample 48971 (Bird B). Results provide each identified known and novel species associated with their bin number. Completeness percentage is given as well as marker gene overrepresentation, novelty score, abundance, contig N50, genome size, number of contigs, and GC percentage of each isolate. Table S3. PHASTEST results of phage elements associated with sample 13009 and 48971 isolates categorized as intact, questionable, or incomplete. Figure S1. Analysis of microbes in Bird A and B provided by Phase Genomics’ ProxiMeta Explorer. (A,D) Bin overview of each isolate and its completeness. (B,E) Percentage of abundance within the metagenome by genus and TPM (C,F) Breakdown of microbe presence by genus and TPM, with ring size indicating the level of completeness. Figure S2. Analysis of viral elements in Bird A and B provided by Phase Genomics’ ProxiMeta Explorer. (A,C) Breakdown of virus–host association by genus and TPM. Ring size indicates viral genome size (bp). Squares represent a provirus. (B,D) Percentage of abundance of viral elements by host genus and TPM. Figure S3. Analysis of plasmids in Bird A and B provided by Phase Genomics’ ProxiMeta Explorer. (A,C) Breakdown of plasmid-host association by genus and TPM, with ring size indicating plasmid size (bp). (B,D) Percentage of abundance of plasmids within the fecal sample by host genus and TPM.
