# Supplementary material for: Hi-C Metagenome Deconvolution of Double-Crested Cormorant (Nannopterum auritum) Fecal Samples Demonstrates Feasibility of Linking Microbial Genomes, AMR Genes, and Mobile Elements in Avian Microbiomes
Source: Microorganisms. 2026 May 26;14(6):1198. doi: 10.3390/microorganisms14061198 (PMC13303754; doi:10.3390/microorganisms14061198)
Supplement: Supplementary file 1 [file microorganisms-14-01198-s001.zip › Supplemental Information Descriptions DCCO Manuscript.pdf]

Supplemental Information

Table S1. Phase Genomics Hi-C ProxiMeta results of cormorant fecal sample 13009. Results provide each identified known and novel species associated with their bin number. Completeness percentage is given as well as marker gene overrepresentation, novelty score, abundance, contig N50, genome size, number of contigs and GC percentage of each isolate.

Table S2. Phase Genomics Hi-C ProxiMeta results of cormorant fecal sample 48971. Results provide each identified known and novel species associated with their bin number. Completeness percentage is given as well as marker gene overrepresentation, novelty score, abundance, contig N50, genome size, number of contigs and GC percentage of each isolate.

Table S3. PHASTEST results of phage elements associated with sample 13009 and 48971 isolates categorized as intact, questionable, or incomplete.

Figure S1. Analysis of microbes in samples 13009 and 48971 provided by Phase Genomics' ProxiMeta Explorer. **(A, D)** Bin overview of each isolate and its completeness. **(B, E)** Percentage of abundance within the metagenome by genus and RPKM **(C, F)** Breakdown of microbe presence by genus and RPKM, with ring size indicating the level of completeness.

Figure S2. Analysis of viral elements in samples 13009 and 48971 provided by Phase Genomics' ProxiMeta Explorer. **(A, C)** Breakdown of virus-host association by genus and RPKM. Ring size indicates viral genome size (bp). Squares represent a provirus. **(B, D)** Percentage of abundance of viral elements by host genus and RPKM.

Figure S3. Analysis of plasmids in samples 13009 and 48971 provided by Phase Genomics' ProxiMeta Explorer. (A, C) Breakdown of plasmid-host association by genus and RPKM, with ring size indicating plasmid size (bp). (B, D) Percentage of abundance of plasmids within the fecal sample by host genus and RPKM.
