# Supplementary material for: Hi-C Metagenome Deconvolution of Double-Crested Cormorant (Nannopterum auritum) Fecal Samples Demonstrates Feasibility of Linking Microbial Genomes, AMR Genes, and Mobile Elements in Avian Microbiomes
Source: Microorganisms. 2026 May 26;14(6):1198. doi: 10.3390/microorganisms14061198 (PMC13303754; doi:10.3390/microorganisms14061198)
Supplement: Supplementary file 1 [file microorganisms-14-01198-s001.zip › TableS1.pdf]

Results

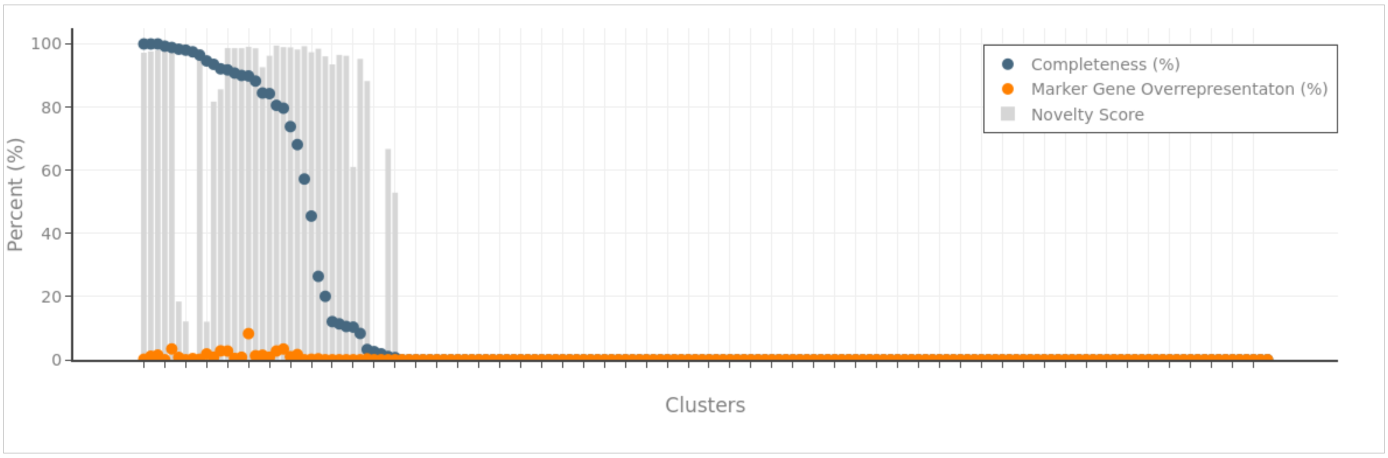

Novel Genome
>70% Complete, <10% MGO*
>90 Novelty Score

Known Genome
>70% Complete, <10% MGO*
<90 Novelty Score

*Marker Gene Overrepresentation

Cluster ID	Top Reference	Complete (%)	MGO (%)	Novelty Score	Abundance[1]	Contig N50	Genome Size	Num Contigs	GC (%)
bin_6	*k__Bacteria (UID2329)*	100.00	0.16	97.23	1.09	27,226	2,586,251	64	28.36
bin_7	*k__Bacteria (UID2329)*	100.00	1.12	97.62	0.40	163,501	2,200,486	120	28.23
bin_11	*p__Firmicutes (UID1022)*	100.00	1.48	98.01	0.08	97,977	1,901,298	34	34.77
bin_12	*o__Clostridiales (UID1120)*	99.30	0.00	98.80	0.06	51,471	1,894,079	84	30.59
bin_14	*k__Bacteria (UID2329)*	98.88	3.42	97.59	0.27	21,191	1,823,829	173	28.20
bin_23	Catellicoccus_marimammalium_M35_04_3	98.34	0.74	18.44	0.13	1,023,821	1,367,748	15	33.70
bin_21	Campylobacter_volucris_LMG_24379	98.05	0.00	12.12	2.93	77,015	1,506,994	48	28.17
bin_3	Edwardsiella_tarda_ATCC_15947_NBRC_105688	97.51	0.36	0.32	1.51	782,853	3,840,500	33	56.75
bin_18	*o__Selenomonadales (UID1024)*	96.47	0.15	98.35	0.09	56,858	1,622,822	78	38.32
bin_5	Plesiomonas_shigelloides	94.63	1.84	12.03	0.46	10,168	3,268,833	373	51.55
bin_4	*o__Clostridiales (UID1375)*	93.55	0.81	81.74	0.11	123,993	3,295,749	59	27.58
bin_8	*k__Bacteria (UID2329)*	92.13	2.81	85.65	0.26	6,250	2,139,963	330	31.17
bin_20	*c__Epsilonproteobacteria (UID3066)*	91.79	2.75	98.70	2.89	50,597	1,514,045	69	29.86
bin_19	*c__Epsilonproteobacteria (UID3066)*	90.79	0.43	98.68	1.32	118,224	1,550,314	50	36.74
bin_10	*f__Porphyromonadaceae (UID2622)*	90.03	0.79	98.67	0.51	6,232	2,017,534	257	33.74
bin_13	*c__Epsilonproteobacteria (UID3066)*	89.87	8.27	99.11	8.79	33,637	1,849,317	170	54.60
bin_22	*o__Clostridiales (UID1226)*	88.25	1.27	98.65	0.34	5,139	1,406,458	183	28.65
bin_9	*o__Actinomycetales (UID1590)*	84.47	1.45	92.64	0.18	8,432	2,120,299	355	74.34
bin_29	*o__Campylobacterales (UID3068)*	84.27	0.82	96.26	0.01	249,928	1,248,954	9	35.79
bin_28	*c__Epsilonproteobacteria (UID3066)*	80.56	2.77	99.50	0.17	4,948	1,263,334	220	39.88
bin_17	*c__Epsilonproteobacteria (UID3066)*	79.66	3.40	99.00	1.71	15,913	1,686,941	297	54.10
bin_24	*f__Actinomycetaceae (UID1531)*	73.83	0.98	98.92	0.24	9,127	1,355,230	272	49.96
bin_33	*o__Clostridiales (UID1212)*	68.13	1.65	98.25	0.13	4,583	980,876	202	29.02
bin_25	*k__Bacteria (UID203)*	57.23	0.00	99.30	0.26	5,567	1,346,369	212	57.32
bin_39	*p__Proteobacteria (UID3887)*	45.50	0.16	97.39	0.02	4,608	701,018	105	52.09
bin_64	*f__Actinomycetaceae (UID1531)*	26.41	0.29	98.49	0.05	10,058	379,475	112	48.83
bin_77	*k__Bacteria (UID2329)*	20.08	0.00	96.08	0.06	2,454	282,661	105	25.59
bin_94	*k__Bacteria (UID203)*	12.07	0.00	93.53	0.03	2,828	208,075	63	28.85
bin_30	*k__Archaea (UID2)*	11.36	0.00	96.53	0.03	8,028	1,209,751	324	37.37
bin_112	*k__Bacteria (UID203)*	10.53	0.00	96.26	0.00	107,232	173,151	4	39.36
bin_54	*k__Bacteria (UID203)*	10.34	0.00	61.05	0.01	37,364	533,461	62	27.39
bin_56	*root (UID1)*	8.33	0.00	95.28	0.06	255,943	515,731	72	27.64
bin_151	*c__Epsilonproteobacteria (UID3066)*	3.25	0.27	88.25	0.02	7,001	108,506	39	30.12
bin_1	*k__Bacteria (UID203)*	2.57	0.00	0.00	3.61	2,914	46,676,931	5681	56.38
bin_2	*k__Bacteria (UID203)*	1.88	0.00	1.71	1.98	3,942	27,653,251	5502	51.05
bin_26	*root (UID1)*	1.04	0.00	66.74	0.12	7,563	1,323,780	136	53.17
bin_53	*k__Archaea (UID2)*	0.68	0.00	52.93	0.15	11,383	548,851	62	49.11
bin_15	*root (UID1)*	0.00	0.00	0.00	0.11	3,587	1,813,432	460	56.64
bin_16	*root (UID1)*	0.00	0.00	0.00	0.11	7,055	1,728,911	313	69.43
bin_27	*root (UID1)*	0.00	0.00	0.00	0.11	12,127	1,312,353	129	55.03
bin_31	*root (UID1)*	0.00	0.00	0.00	0.10	19,033	1,190,994	116	57.31

Cluster ID	Top Reference	Complete (%)	MGO (%)	Novelty Score	Abundance	Contig N50	Genome Size	Num Contigs	GC (%)
bin_32	*root (UID1)*	0.00	0.00	0.00	0.07	21,112	1,072,132	149	65.44
bin_34	*root (UID1)*	0.00	0.00	0.00	0.07	6,085	947,559	94	58.43
bin_35	*root (UID1)*	0.00	0.00	0.00	0.07	6,950	936,609	97	60.48
bin_36	*root (UID1)*	0.00	0.00	0.00	0.06	20,959	813,253	82	51.62
bin_37	*root (UID1)*	0.00	0.00	0.00	0.06	25,227	714,563	78	53.42
bin_38	*root (UID1)*	0.00	0.00	0.00	1.35	11,193	710,167	133	52.32
bin_40	*root (UID1)*	0.00	0.00	0.00	0.05	12,703	676,745	71	53.39
bin_41	*root (UID1)*	0.00	0.00	0.00	0.06	15,615	666,303	74	52.14
bin_42	*root (UID1)*	0.00	0.00	0.00	0.05	2,890	661,282	78	52.17
bin_43	*root (UID1)*	0.00	0.00	0.00	0.06	19,369	650,179	60	53.39
bin_44	*root (UID1)*	0.00	0.00	0.00	0.05	11,295	645,611	81	51.60
bin_45	*root (UID1)*	0.00	0.00	0.00	0.05	6,373	620,568	70	55.31
bin_46	*root (UID1)*	0.00	0.00	0.00	0.06	22,448	611,912	54	53.12
bin_47	*root (UID1)*	0.00	0.00	0.00	0.05	4,262	603,387	66	51.18
bin_48	*root (UID1)*	0.00	0.00	0.00	0.05	14,144	585,047	53	53.36
bin_49	*root (UID1)*	0.00	0.00	0.00	0.04	34,111	583,080	56	52.30
bin_50	*root (UID1)*	0.00	0.00	0.00	0.04	8,696	573,025	57	53.62
bin_51	*root (UID1)*	0.00	0.00	0.00	0.07	15,833	568,760	75	48.84
bin_52	*root (UID1)*	0.00	0.00	0.00	0.06	9,573	567,795	60	58.11
bin_55	*root (UID1)*	0.00	0.00	0.00	0.04	4,527	521,117	59	52.62
bin_57	*root (UID1)*	0.00	0.00	0.00	0.10	12,943	506,506	58	52.68
bin_58	*root (UID1)*	0.00	0.00	0.00	0.04	3,096	501,443	54	56.27
bin_59	*root (UID1)*	0.00	0.00	0.00	0.04	38,452	474,963	45	51.18
bin_60	*root (UID1)*	0.00	0.00	0.00	0.04	17,382	463,106	50	53.53
bin_61	*root (UID1)*	0.00	0.00	0.00	0.03	5,004	452,144	48	56.78
bin_62	*root (UID1)*	0.00	0.00	0.00	0.03	8,084	435,234	46	53.43
bin_63	*root (UID1)*	0.00	0.00	0.00	0.03	7,560	434,921	58	54.90
bin_65	*root (UID1)*	0.00	0.00	0.00	0.03	15,741	372,618	40	60.74
bin_66	*root (UID1)*	0.00	0.00	0.00	0.03	6,927	365,998	41	52.77
bin_67	*root (UID1)*	0.00	0.00	0.00	0.03	7,869	346,084	39	50.39
bin_68	*root (UID1)*	0.00	0.00	0.00	0.03	6,317	329,335	33	51.55
bin_69	*root (UID1)*	0.00	0.00	0.00	0.02	5,304	325,539	76	65.10
bin_70	*root (UID1)*	0.00	0.00	0.00	0.02	8,800	314,473	35	52.16
bin_71	*root (UID1)*	0.00	0.00	0.00	0.02	21,496	307,480	33	56.71
bin_72	*root (UID1)*	0.00	0.00	0.00	0.02	21,891	305,363	28	53.18
bin_73	*root (UID1)*	0.00	0.00	0.00	0.02	12,973	304,675	28	52.99
bin_74	*root (UID1)*	0.00	0.00	0.00	0.02	11,864	293,954	33	50.79
bin_75	*root (UID1)*	0.00	0.00	0.00	0.02	2,081	293,839	34	52.01
bin_76	*root (UID1)*	0.00	0.00	0.00	0.02	4,596	285,250	29	61.01
bin_78	*root (UID1)*	0.00	0.00	0.00	0.02	13,140	279,582	27	53.49
bin_79	*root (UID1)*	0.00	0.00	0.00	0.02	2,632	275,787	32	53.39
bin_80	*root (UID1)*	0.00	0.00	0.00	0.02	13,341	266,245	25	52.90
bin_81	*root (UID1)*	0.00	0.00	0.00	0.02	57,154	266,101	21	53.73
bin_82	*root (UID1)*	0.00	0.00	0.00	0.02	7,276	264,208	36	50.51
bin_83	*root (UID1)*	0.00	0.00	0.00	0.02	4,115	260,733	75	63.02
bin_84	*root (UID1)*	0.00	0.00	0.00	0.01	2,785	256,728	66	25.14
bin_85	*root (UID1)*	0.00	0.00	0.00	0.02	14,377	238,116	30	55.97
bin_86	*root (UID1)*	0.00	0.00	0.00	0.02	7,771	225,897	29	49.62
bin_87	*root (UID1)*	0.00	0.00	0.00	0.02	10,332	224,678	24	50.79
bin_88	*root (UID1)*	0.00	0.00	0.00	0.02	6,595	219,238	22	49.20
bin_89	*root (UID1)*	0.00	0.00	0.00	0.02	36,663	218,996	20	52.89
bin_90	*root (UID1)*	0.00	0.00	0.00	0.02	11,578	218,644	28	50.51
bin_91	*root (UID1)*	0.00	0.00	0.00	0.02	11,070	214,751	22	50.57
bin_92	*root (UID1)*	0.00	0.00	0.00	0.02	15,470	212,131	21	52.79
bin_93	*root (UID1)*	0.00	0.00	0.00	0.02	18,481	211,303	26	48.47
bin_95	*root (UID1)*	0.00	0.00	0.00	0.02	2,315	204,975	21	53.07
bin_96	*root (UID1)*	0.00	0.00	0.00	0.02	4,330	200,214	32	48.74
bin_97	*root (UID1)*	0.00	0.00	0.00	0.01	14,183	193,792	21	51.92
bin_98	*root (UID1)*	0.00	0.00	0.00	0.01	2,007	192,775	27	49.09
bin_99	*root (UID1)*	0.00	0.00	0.00	0.03	3,566	187,015	25	54.87

Cluster ID	Top Reference	Complete (%)	MGO (%)	Novelty Score	Abundance	Contig N50	Genome Size	Num Contigs	GC (%)
bin_100	*root (UID1)*	0.00	0.00	0.00	0.04	8,852	186,995	20	49.65
bin_101	*root (UID1)*	0.00	0.00	0.00	0.01	9,267	182,594	16	54.29
bin_102	*root (UID1)*	0.00	0.00	0.00	0.01	6,725	181,704	23	59.67
bin_103	*root (UID1)*	0.00	0.00	0.00	0.01	7,005	181,371	13	53.28
bin_104	*root (UID1)*	0.00	0.00	0.00	0.01	4,411	180,861	25	48.09
bin_105	*root (UID1)*	0.00	0.00	0.00	0.01	10,048	180,424	20	50.74
bin_106	*root (UID1)*	0.00	0.00	0.00	0.01	16,669	179,741	24	47.65
bin_107	*root (UID1)*	0.00	0.00	0.00	0.02	9,250	178,645	21	53.38
bin_108	*root (UID1)*	0.00	0.00	0.00	0.01	4,303	177,879	19	51.05
bin_109	*root (UID1)*	0.00	0.00	0.00	0.01	21,791	176,209	17	53.62
bin_110	*root (UID1)*	0.00	0.00	0.00	0.01	7,844	175,424	20	55.47
bin_111	*root (UID1)*	0.00	0.00	0.00	0.01	32,816	174,718	19	53.90
bin_113	*root (UID1)*	0.00	0.00	0.00	0.01	19,247	165,820	21	47.89
bin_114	*root (UID1)*	0.00	0.00	0.00	0.01	8,237	165,663	22	55.87
bin_115	*root (UID1)*	0.00	0.00	0.00	0.01	10,921	164,685	19	53.79
bin_116	*root (UID1)*	0.00	0.00	0.00	0.02	13,346	161,857	21	52.06
bin_117	*root (UID1)*	0.00	0.00	0.00	0.02	11,073	161,856	20	49.85
bin_118	*root (UID1)*	0.00	0.00	0.00	0.01	14,366	154,765	19	54.73
bin_119	*root (UID1)*	0.00	0.00	0.00	0.01	8,614	148,732	24	47.69
bin_120	*root (UID1)*	0.00	0.00	0.00	0.01	13,827	148,008	20	50.73
bin_121	*root (UID1)*	0.00	0.00	0.00	0.01	3,858	147,884	20	51.39
bin_122	*root (UID1)*	0.00	0.00	0.00	0.01	3,633	144,902	19	53.47
bin_123	*root (UID1)*	0.00	0.00	0.00	0.01	3,135	144,647	16	47.30
bin_124	*root (UID1)*	0.00	0.00	0.00	0.01	5,093	143,809	21	49.87
bin_125	*root (UID1)*	0.00	0.00	0.00	0.01	8,922	142,039	22	48.01
bin_126	*root (UID1)*	0.00	0.00	0.00	0.01	3,538	136,693	20	50.32
bin_127	*root (UID1)*	0.00	0.00	0.00	0.01	4,330	136,188	18	51.22
bin_128	*root (UID1)*	0.00	0.00	0.00	0.01	23,722	135,475	10	55.70
bin_129	*root (UID1)*	0.00	0.00	0.00	0.01	3,883	134,184	17	49.85
bin_130	*root (UID1)*	0.00	0.00	0.00	0.01	17,844	133,519	19	50.16
bin_131	*root (UID1)*	0.00	0.00	0.00	0.01	5,909	132,102	21	48.09
bin_132	*root (UID1)*	0.00	0.00	0.00	0.01	10,629	131,826	26	46.26
bin_133	*root (UID1)*	0.00	0.00	0.00	0.01	8,534	131,156	14	52.02
bin_134	*root (UID1)*	0.00	0.00	0.00	0.01	7,334	131,078	20	49.02
bin_135	*root (UID1)*	0.00	0.00	0.00	0.01	9,109	130,701	15	53.24
bin_136	*root (UID1)*	0.00	0.00	0.00	0.01	16,142	129,149	15	64.02
bin_137	*root (UID1)*	0.00	0.00	0.00	0.01	14,600	129,133	15	51.82
bin_138	*root (UID1)*	0.00	0.00	0.00	0.01	39,394	129,040	15	52.66
bin_139	*root (UID1)*	0.00	0.00	0.00	0.01	7,004	128,493	10	54.86
bin_140	*root (UID1)*	0.00	0.00	0.00	0.01	5,308	126,017	17	49.25
bin_141	*root (UID1)*	0.00	0.00	0.00	0.01	8,662	121,933	26	59.71
bin_142	*root (UID1)*	0.00	0.00	0.00	0.01	3,074	121,129	30	70.47
bin_143	*root (UID1)*	0.00	0.00	0.00	0.01	8,702	119,734	18	46.87
bin_144	*root (UID1)*	0.00	0.00	0.00	0.01	31,642	114,801	11	66.82
bin_145	*root (UID1)*	0.00	0.00	0.00	0.01	18,064	113,399	15	52.84
bin_146	*root (UID1)*	0.00	0.00	0.00	0.01	5,349	113,252	11	42.40
bin_147	*root (UID1)*	0.00	0.00	0.00	0.02	14,173	111,211	14	42.96
bin_148	*root (UID1)*	0.00	0.00	0.00	0.01	26,809	109,897	11	54.88
bin_149	*root (UID1)*	0.00	0.00	0.00	0.01	27,237	109,754	15	48.18
bin_150	*root (UID1)*	0.00	0.00	0.00	0.01	7,232	108,585	14	52.71
bin_152	*root (UID1)*	0.00	0.00	0.00	0.01	5,289	106,568	16	49.41
bin_153	*root (UID1)*	0.00	0.00	0.00	0.01	6,049	105,857	15	49.30
bin_154	*root (UID1)*	0.00	0.00	0.00	0.01	4,611	104,268	16	48.05
bin_155	*root (UID1)*	0.00	0.00	0.00	0.01	11,267	102,968	11	49.70
bin_156	*root (UID1)*	0.00	0.00	0.00	0.00	3,222	102,384	33	31.65
bin_157	*root (UID1)*	0.00	0.00	0.00	0.01	7,522	101,911	13	59.17
bin_158	*root (UID1)*	0.00	0.00	0.00	0.01	13,376	101,755	13	51.89
bin_159	*root (UID1)*	0.00	0.00	0.00	0.01	32,012	101,726	10	55.46
bin_160	*root (UID1)*	0.00	0.00	0.00	0.01	11,312	101,017	13	47.08
bin_161	*root (UID1)*	0.00	0.00	0.00	0.01	14,168	101,016	13	51.46

Cluster ID	Top Reference	Complete (%)	MGO (%)	Novelty Score	Abundance	Contig N50	Genome Size	Num Contigs	GC (%)
bin_162	*root (UID1)*	0.00	0.00	0.00	0.01	7,069	100,360	15	48.97

1) For samples processed after December 2, 2020 abundance will show the average read depth for each bin by length. Older samples will show an estimated relative abundance percentage.
