# Supplementary material for: Hi-C Metagenome Deconvolution of Double-Crested Cormorant (Nannopterum auritum) Fecal Samples Demonstrates Feasibility of Linking Microbial Genomes, AMR Genes, and Mobile Elements in Avian Microbiomes
Source: Microorganisms. 2026 May 26;14(6):1198. doi: 10.3390/microorganisms14061198 (PMC13303754; doi:10.3390/microorganisms14061198)
Supplement: Supplementary file 1 [file microorganisms-14-01198-s001.zip › TableS2.pdf]

PHASE GENOMICS

KEEN-48971
01/21/2022

Results

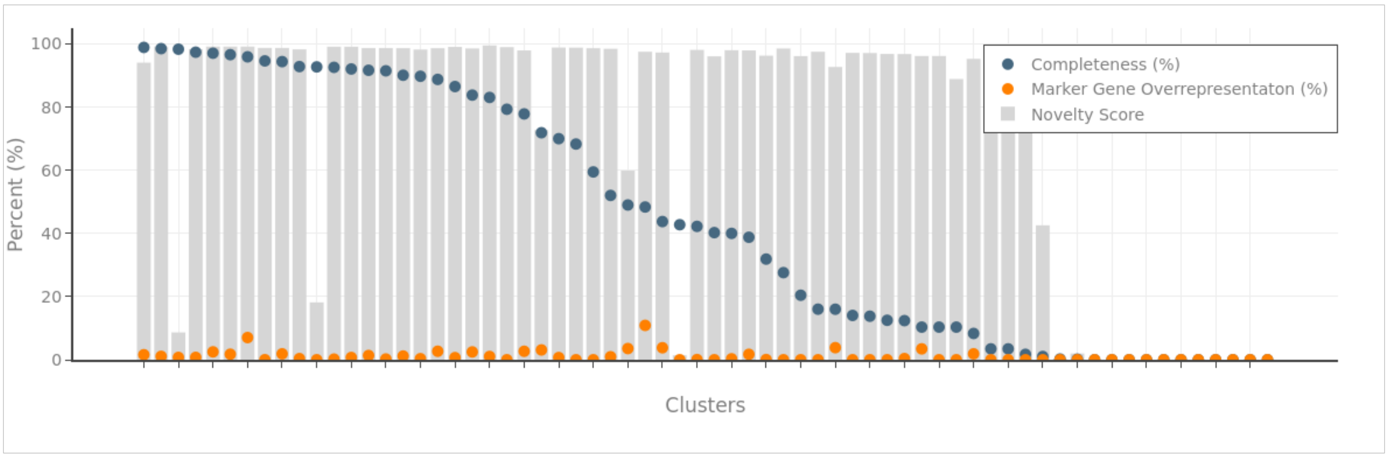

Novel Genome
>70% Complete, <10% MGO*
>90 Novelty Score

Known Genome
>70% Complete, <10% MGO*
<90 Novelty Score

*Marker Gene Overrepresentation

Cluster ID	Top Reference	Complete (%)	MGO (%)	Novelty Score	Abundance[1]	Contig N50	Genome Size	Num Contigs	GC (%)
bin_8	*o__Actinomycetales (UID1590)*	98.91	1.59	94.07	0.22	71,588	2,609,314	89	74.79
bin_7	*o__Actinomycetales (UID1590)*	98.53	1.08	99.19	0.18	467,041	2,666,558	28	60.04
bin_29	Catellicoccus_marimammalium_M35_04_3	98.34	0.74	8.66	0.01	1,015,554	1,301,184	6	33.82
bin_11	*o__Actinomycetales (UID1809)*	97.40	0.75	98.37	0.07	87,253	2,135,811	55	60.98
bin_10	*p__Actinobacteria (UID1454)*	97.08	2.50	99.18	0.11	22,620	2,209,776	128	61.43
bin_17	*f__Actinomycetaceae (UID1531)*	96.61	1.75	99.17	0.04	13,823	1,775,997	38	48.51
bin_9	*f__Actinomycetaceae (UID1531)*	95.93	7.03	99.17	0.23	97,809	2,432,383	182	60.53
bin_12	*o__Clostridiales (UID1212)*	94.64	0.02	98.74	0.57	248,518	2,055,247	116	25.79
bin_16	*f__Micrococcaceae (UID1623)*	94.41	1.87	98.73	0.03	16,276	1,869,592	71	57.03
bin_13	*o__Clostridiales (UID1212)*	92.84	0.36	98.29	0.05	26,283	2,026,667	52	24.32
bin_6	Clostridium_perfringens_ATCC_13124	92.74	0.00	18.16	0.02	411,685	2,773,671	28	27.91
bin_22	*o__Clostridiales (UID1125)*	92.60	0.20	99.14	0.06	11,250	1,556,095	54	46.34
bin_23	*o__Clostridiales (UID1120)*	92.08	0.71	99.13	0.06	21,711	1,549,620	88	56.41
bin_21	*o__Selenomonadales (UID1024)*	91.67	1.35	98.70	0.06	4,283	1,559,231	136	38.22
bin_18	*o__Clostridiales (UID1212)*	91.50	0.23	98.69	0.17	121,991	1,721,280	65	27.44
bin_14	*o__Actinomycetales (UID1697)*	90.10	1.18	98.67	0.03	20,360	1,975,032	162	61.58
bin_20	*c__Betaproteobacteria (UID3959)*	89.78	0.33	98.23	0.04	15,239	1,593,082	142	41.51
bin_15	*o__Actinomycetales (UID1812)*	88.78	2.69	98.66	0.05	13,413	1,973,127	190	62.05
bin_31	*o__Clostridiales (UID1212)*	86.52	0.67	99.08	0.03	4,390	1,152,148	100	28.85
bin_19	*o__Clostridiales (UID1120)*	83.81	2.48	98.58	0.05	91,195	1,691,311	57	23.38
bin_25	*c__Epsilonproteobacteria (UID3066)*	83.07	1.04	99.52	0.15	30,502	1,475,623	93	50.27
bin_38	*k__Bacteria (UID2328)*	79.33	0.00	99.00	0.01	96,073	748,216	19	26.56
bin_26	*o__Clostridiales (UID1212)*	77.85	2.68	97.96	0.04	18,116	1,461,644	223	25.20
bin_2	Phalacrocorax_carbo	71.89	3.11	72.73	5.30	32,503	78,326,830	3728	41.03
bin_24	*o__Actinomycetales (UID1697)*	70.01	0.69	98.86	0.02	12,746	1,531,603	229	58.88
bin_32	*o__Clostridiales (UID1120)*	68.31	0.00	98.83	0.01	22,967	1,133,747	32	28.13
bin_27	*k__Bacteria (UID203)*	59.48	0.00	98.66	0.04	21,726	1,359,592	89	39.47
bin_34	*f__Actinomycetaceae (UID1531)*	52.04	0.95	98.47	0.02	39,500	898,109	43	49.13
bin_1	Phalacrocorax_carbo	49.01	3.55	59.87	5.76	39,307	84,761,051	3900	41.13
bin_3	*k__Archaea (UID2)*	48.37	10.88	97.54	0.78	11,309	8,886,236	1648	35.57
bin_33	*p__Firmicutes (UID1022)*	43.76	3.80	97.29	0.02	16,830	909,843	167	29.80
bin_39	Lactococcus_garvieae_ATCC_49156	42.76	0.00	1.42	0.01	5,890	731,425	117	39.63
bin_37	*c__Epsilonproteobacteria (UID3066)*	42.24	0.05	98.12	0.11	11,659	775,867	144	58.27
bin_43	*c__Epsilonproteobacteria (UID3066)*	40.26	0.00	96.08	0.02	40,549	578,396	28	29.54
bin_35	*o__Actinomycetales (UID1572)*	40.02	0.29	98.02	0.01	4,552	846,721	96	65.29
bin_41	*k__Bacteria (UID203)*	38.79	1.72	97.95	0.04	18,626	674,652	67	40.04
bin_40	*c__Epsilonproteobacteria (UID3066)*	31.88	0.06	96.29	0.26	31,357	709,838	14	32.98
bin_42	*k__Bacteria (UID203)*	27.59	0.00	98.56	0.02	11,855	585,893	35	46.61
bin_44	*o__Clostridiales (UID1125)*	20.43	0.00	96.14	0.00	35,043	354,415	16	29.50
bin_45	*k__Bacteria (UID203)*	16.02	0.00	97.53	0.01	12,334	352,879	39	47.91
bin_4	*k__Archaea (UID2)*	16.00	3.83	92.71	0.18	2,935	4,972,797	1364	28.18

Cluster ID	Top Reference	Complete (%)	MGO (%)	Novelty Score	Abundance	Contig N50	Genome Size	Num Contigs	GC (%)
bin_46	*k__Bacteria (UID203)*	14.04	0.00	97.18	0.01	25,132	346,650	18	61.69
bin_53	*k__Bacteria (UID203)*	13.79	0.00	97.13	0.01	8,304	211,489	22	48.46
bin_49	*root (UID1)*	12.50	0.00	96.84	0.01	11,557	320,056	33	50.99
bin_57	*c__Epsilonproteobacteria (UID3066)*	12.39	0.37	96.81	0.01	23,058	186,275	16	45.12
bin_52	*k__Bacteria (UID203)*	10.34	3.45	96.19	0.00	13,588	245,361	29	41.33
bin_55	*k__Bacteria (UID203)*	10.34	0.00	96.19	0.00	11,827	204,937	30	53.03
bin_59	*k__Bacteria (UID203)*	10.34	0.00	88.89	0.00	23,924	161,588	6	29.39
bin_30	*k__Archaea (UID2)*	8.33	1.87	95.28	0.08	3,214	1,168,896	294	33.63
bin_48	*k__Bacteria (UID203)*	3.45	0.00	78.75	0.01	3,331	334,578	92	32.91
bin_60	*k__Bacteria (UID203)*	3.45	0.00	88.90	0.00	13,766	155,051	14	50.24
bin_58	*k__Bacteria (UID203)*	1.72	0.00	78.69	0.02	6,225	177,052	29	55.59
bin_5	*root (UID1)*	1.04	0.00	42.56	0.36	6,976	3,041,074	89	48.54
bin_64	*k__Archaea (UID2)*	0.22	0.00	0.01	0.01	4,686	128,009	13	22.00
bin_54	*k__Bacteria (UID203)*	0.16	0.00	1.98	0.01	205,880	205,880	1	28.28
bin_28	*root (UID1)*	0.00	0.00	0.00	0.13	100,229	1,353,821	34	47.65
bin_36	*root (UID1)*	0.00	0.00	0.00	0.08	86,237	797,612	29	48.22
bin_47	*root (UID1)*	0.00	0.00	0.00	0.03	7,165	343,184	27	47.98
bin_50	*root (UID1)*	0.00	0.00	0.00	0.05	5,209	316,710	37	56.32
bin_51	*root (UID1)*	0.00	0.00	0.00	0.03	52,436	292,196	22	46.69
bin_56	*root (UID1)*	0.00	0.00	0.00	0.02	3,149	201,580	37	59.24
bin_61	*root (UID1)*	0.00	0.00	0.00	0.00	2,544	154,482	51	23.44
bin_62	*root (UID1)*	0.00	0.00	0.00	0.00	73,298	144,935	4	47.89
bin_63	*root (UID1)*	0.00	0.00	0.00	0.00	57,072	139,954	3	50.37
bin_65	*root (UID1)*	0.00	0.00	0.00	0.01	2,577	119,641	31	61.87
bin_66	*root (UID1)*	0.00	0.00	0.00	0.01	2,800	107,802	27	51.99

1) For samples processed after December 2, 2020 abundance will show the average read depth for each bin by length. Older samples will show an estimated relative abundance percentage.
