# Supplementary figures and images for: Hi-C Metagenome Deconvolution of Double-Crested Cormorant (Nannopterum auritum) Fecal Samples Demonstrates Feasibility of Linking Microbial Genomes, AMR Genes, and Mobile Elements in Avian Microbiomes
Source: Microorganisms. 2026 May 26;14(6):1198. doi: 10.3390/microorganisms14061198 (PMC13303754; doi:10.3390/microorganisms14061198)

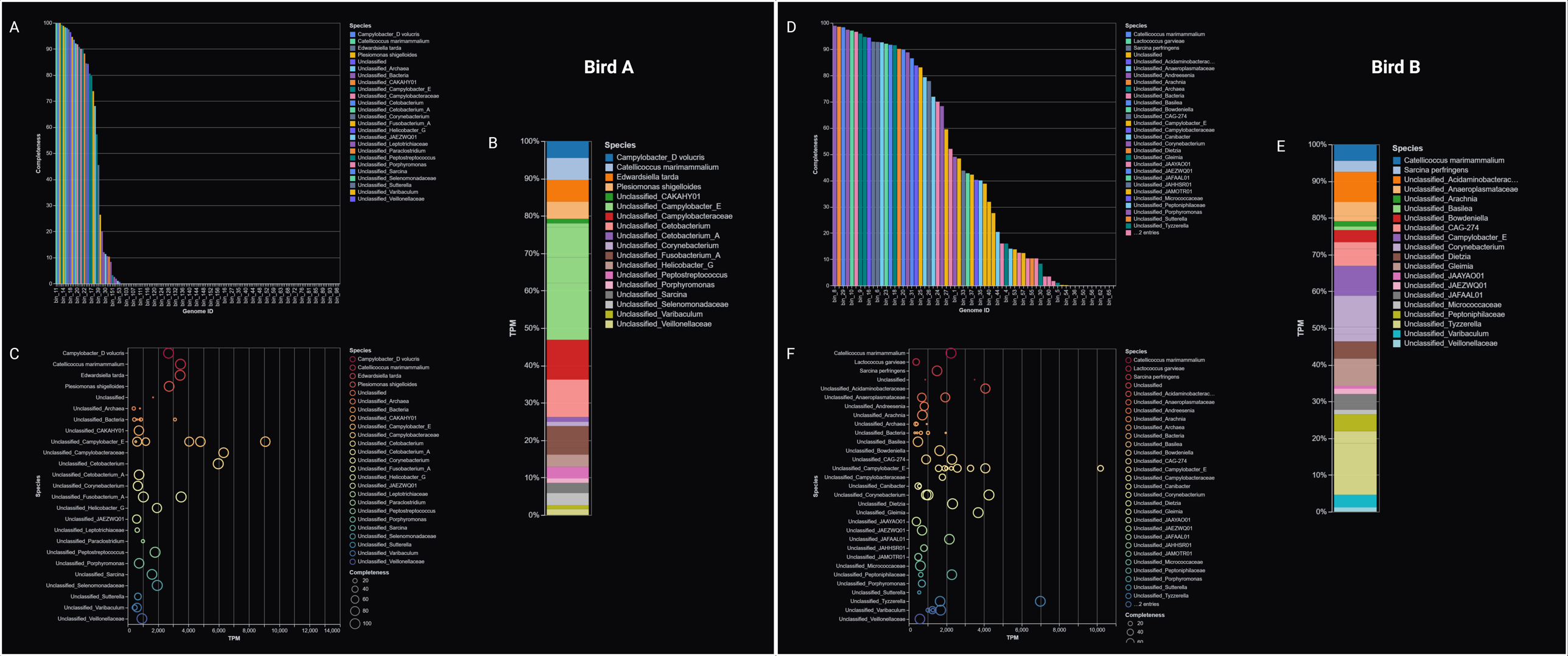

Supplement: Supplementary file 1 [file microorganisms-14-01198-s001.zip › FigureS1.tiff]

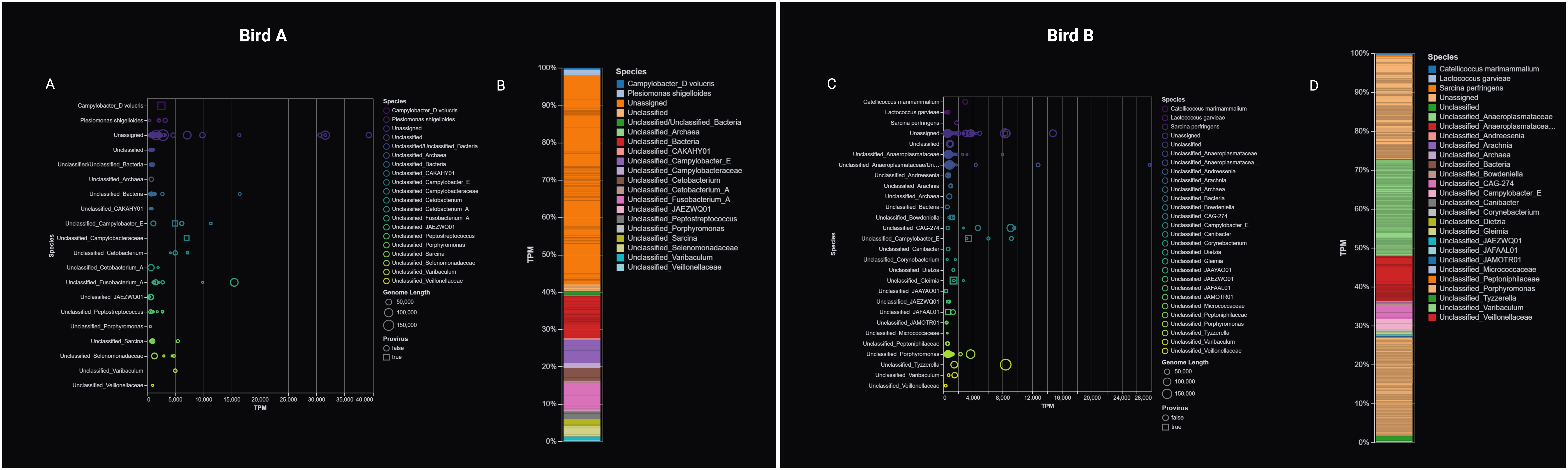

Supplement: Supplementary file 1 [file microorganisms-14-01198-s001.zip › FigureS2.tiff]

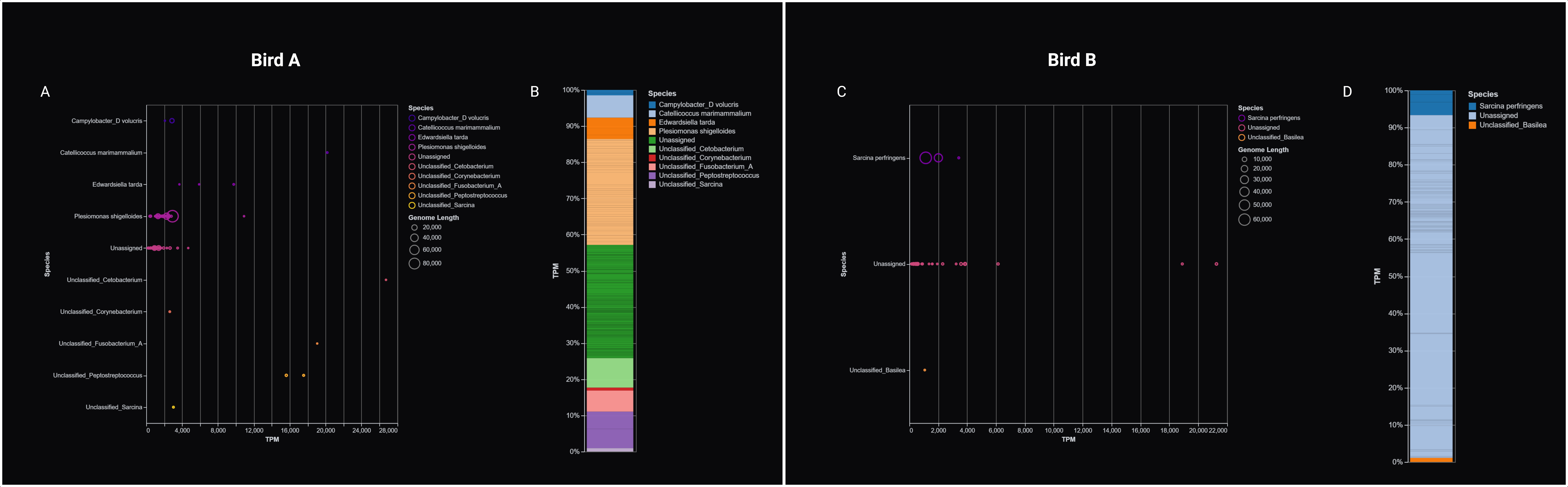

Supplement: Supplementary file 1 [file microorganisms-14-01198-s001.zip › FigureS3.tiff]
